# Cellulose and Its Derivatives in Drug Delivery: Recent Advances and Applications

**DOI:** 10.3390/pharmaceutics18050594

**Published:** 2026-05-12

**Authors:** Dan Luo, Yu Wang, Dan Zhou, Shiyan Wang, Mengran Guo

**Affiliations:** Department of Pharmacy, West China Hospital, Sichuan University, Chengdu 610041, China; ldan533@163.com (D.L.); yuu1999@163.com (Y.W.); wang_sy95@scu.edu.cn (S.W.)

**Keywords:** cellulose, cellulose derivatives, nanocellulose, hydrogels, aerogels, films, drug delivery

## Abstract

Drug delivery systems have long faced a fundamental challenge: achieving high drug-loading efficiency, precise control over release, and in vivo safety simultaneously is a difficult task. Cellulose and its derivatives are abundant and renewable, exhibiting good biocompatibility, which makes them promising candidates for drug delivery materials. Representative derivatives, such as carboxymethyl cellulose, hydroxypropyl methyl cellulose, and ethyl cellulose, as well as nanocellulose, including cellulose nanocrystals, cellulose nanofibrils, and bacterial nanocellulose, have enabled the development of diverse carrier formats, including hydrogels, aerogels, films, and particulate systems. Recent advances include pH-responsive bacterial nanocellulose/carboxymethyl cellulose hydrogels for oral ibuprofen delivery, carboxylated nanocellulose/polyethylene glycol/β-cyclodextrin composite aerogels for gastric-selective release of imatinib, and hydroxypropyl methyl cellulose-based microneedle patches for transdermal co-delivery of sumatriptan succinate and naproxen sodium. These examples highlight how cellulose-based systems can be engineered for site-selective delivery, sustained release, and multi-stimuli responsiveness. In this review, we summarize the structural features of cellulose derivatives and nanocellulose, discuss the design principles and release mechanisms of representative delivery platforms, and outline current challenges in manufacturability, safety evaluation, and clinical translation.

## 1. Introduction

The central goal of drug delivery systems (DDS) is to achieve controlled drug exposure in both time and anatomical location within the body under specific administration routes and physiological barriers. This control aims to increase the effective drug concentration and retention at the disease site, while reducing off-target distribution-related toxicity and side effects, and improving patient adherence. In clinical practice, many active pharmaceutical ingredients are constrained by low solubility, poor stability, extensive first-pass metabolism, insufficient tissue penetration, or narrow therapeutic windows. In such cases, simply changing the dose or dosing frequency often cannot balance efficacy and safety. Therefore, identifying material platforms that simultaneously provide high drug-loading capacity, structural and chemical stability, tunable release, in vivo compatibility, and practical manufacturability remains a key focus for DDS research and translation [1].

From a materials perspective, interest in sustainable and bio-based systems has increased steadily in recent years. Cellulose, one of the most abundant renewable polysaccharides in nature, is widely available from plant resources such as wood and cotton, and high-purity bacterial cellulose can also be produced via microbial fermentation [2]. With an established industrial base and cost advantages, cellulose shows strong potential for biomedical use owing to its biocompatibility, low toxicity, and degradability [3,4]. Structurally, cellulose consists of linear polymer chains connected by β-1,4-glycosidic linkages. Extensive intra- and intermolecular hydrogen bonding leads to a semi-crystalline, hierarchical architecture, which underpins its favorable mechanical strength and structural stability. However, the same dense hydrogen-bonding network and crystallinity also result in poor solubility in water and most conventional organic solvents and limit flexibility and processability, which has driven the development of cellulose derivatives and nanocellulose (NC) [2,5].

The abundance of hydroxyl groups and other functionalizable sites on cellulose offers versatile handles for chemical modification. By using hydroxyl groups as reactive sites, cellulose derivatives with distinct properties can be produced. Such derivatives enable the tuning of solubility, swelling behavior, and drug carrier interactions, thereby supporting diffusion-controlled release, adhesion-mediated retention, and stimuli-responsive release [6]. In parallel, NC with a high specific surface area, controllable pore architecture, and nanoscale reinforcement can further enhance drug loading efficiency, mechanical performance, and functional integration [7]. Leveraging these inherent properties, cellulose-based materials can be engineered into diverse carrier formats—including hydrogels, aerogels, and films—whose structural and functional characteristics can be precisely tailored through composite formulation and stimulus-responsive design to fulfill route-specific requirements for controlled drug release kinetics, localized retention, and effective tissue penetration [8,9].

The administration route determines the major barriers and evaluation metrics for a delivery system. For example, oral delivery must cope with gastrointestinal pH gradients, enzymatic degradation, and transport constraints. Transdermal delivery must overcome the stratum corneum barrier while balancing skin irritation and comfort during prolonged application. Owing to its processability and amenability to functionalization, cellulose has gradually enabled diverse design paradigms across these scenarios.

**Figure 1:**
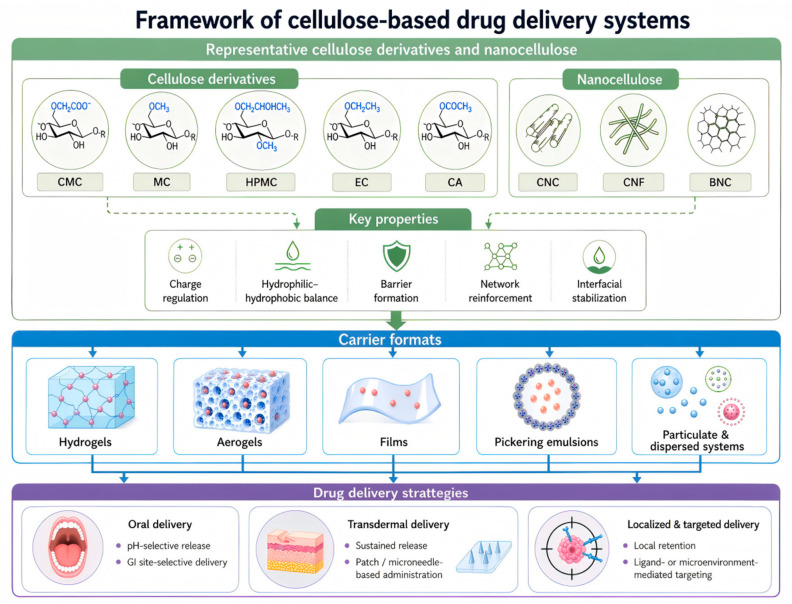
Conceptual framework of cellulose-based drug delivery systems. The figure summarizes the relationships among cellulose materials, carrier formats, delivery functions, and representative application scenarios.

It should be noted that the preparation, modification, and fundamental properties of cellulose and its derivatives, as well as nanocellulose, have already been extensively reviewed. As shown in Figure 1, we focus on the roles that cellulose plays in delivery systems, how controlled delivery can be achieved through structural design and carrier format, and the route-dependent design considerations supported by the available evidence. Within a material, carrier, and application framework, we summarize representative advances and discuss key issues and future directions toward clinical translation, with the aim of providing useful insights for further development of cellulose and its derivatives in drug delivery.

## 2. Representative Cellulose Derivatives and Nanocellulose

### 2.1. Cellulose and Its Derivatives

**Figure 2:**
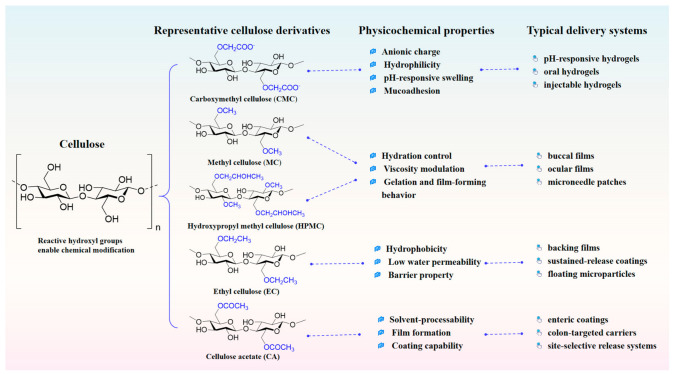
Representative cellulose derivatives, physicochemical properties, and typical delivery systems.

Cellulose is a linear β-1,4-linked polysaccharide whose dense intra- and intermolecular hydrogen-bonding network confers structural stability, while also contributing to poor solubility and limited processability. For drug delivery, the importance of cellulose lies less in these general structural features themselves than in the fact that its abundant hydroxyl groups provide accessible sites for chemical modification. Such modification alters charge, hydrophilic/hydrophobic balance, chain mobility, and solvent compatibility, thereby determining how cellulose-derived materials behave in formulation and which delivery platform they are best suited for [2], as summarized in Figure 2.

From a drug-delivery perspective, representative derivatives can be understood according to the properties they introduce. Carboxymethyl cellulose (CMC) is an anionic and highly hydrophilic derivative whose ionizable carboxymethyl groups support water uptake, mucoadhesion, and pH-responsive swelling, making it particularly useful in hydrogels, oral pH-responsive carriers, and adhesive films. Methylcellulose (MC) and hydroxypropyl methyl cellulose (HPMC) are nonionic cellulose ethers widely used to regulate viscosity, hydration, and gelation behavior; their film-forming ability and, in some cases, thermoresponsive behavior make them attractive for oral films, buccal systems, and microneedle matrices. In contrast, ethyl cellulose (EC) is relatively hydrophobic and is therefore more suitable for diffusion-limiting coatings, backing layers, and sustained-release microparticles. Cellulose acetate and related esters offer improved processability in organic solvent systems and are frequently employed in film coatings, membrane-type systems, and enteric or site-selective release designs [10,11,12,13]. These derivatives therefore reappear across different delivery platforms not as interchangeable materials, but because their substitution patterns lead to distinct transport, barrier, adhesive, or responsive functions.

### 2.2. Nanocellulose

NC further expands the design space by introducing morphology-dependent structural and interfacial functions. NC is commonly classified into cellulose nanocrystals (CNC), cellulose nanofibrils (CNF), and bacterial nanocellulose (BNC) based on differences in aspect ratio, crystallinity, flexibility, and network formation behavior. These attributes are directly relevant to the design and construction of drug delivery carriers [14]. CNCs, because of their rigid rod-like morphology and surface tunability, are especially useful for interfacial stabilization and the assembly of dispersed systems [15]. CNFs, with their high aspect ratio and entangled network-forming capacity, are particularly effective as reinforcing phases in hydrogels, films, and porous matrices, where they can regulate swelling, diffusion pathways, and mechanical integrity [16]. BNC naturally forms highly pure nanofibrillar networks with high water-holding capacity and is therefore well suited for hydrogel, aerogel, and film-based biomedical platforms [17].

In drug delivery, nanocellulose functions less as a generic nanosized material than as a structural and interfacial component that can enhance loading, stabilize multicomponent systems, reinforce carrier mechanics, and improve control over release behavior [7].

## 3. Cellulose-Based Drug Carrier Formats

Cellulose-based drug delivery systems can be organized according to carrier format, because the structure of the carrier largely determines drug-loading behavior, release pathways, and application scenarios. Thereafter, we discuss representative cellulose-based platforms in turn, including hydrogels, aerogels, films, and particulate or dispersed systems, with emphasis on their design features and delivery functions.

### 3.1. Cellulose-Based Hydrogels

Hydrogels are 3D network structures formed by hydrophilic polymer chains. Their construction typically relies on two primary approaches, physical crosslinking and chemical crosslinking. Physical crosslinking relies mainly on noncovalent interactions, including hydrogen bonding, hydrophobic association, electrostatic interactions, and chain entanglement, to build a network. In contrast, chemical crosslinking introduces covalent linkages between polymer chains to create stable junctions, thereby providing more durable structural support and improved shape retention [18]. Their frameworks are typically enriched with hydrophilic groups, including hydroxyl, carboxyl, amino, and sulfonic moieties, which enable them to absorb and retain large amounts of water. In the hydrated state, hydrogels remain soft while still providing mechanical support. Because their porous networks and water-rich microenvironments can partially mimic the structure and hydration of the extracellular matrix, hydrogels often show good biocompatibility and provide interfaces that support cell adhesion, migration, and mass transport [19]. By precisely tuning polymer composition, crosslink density, and crosslinking mode, a wide range of properties can be adjusted, including pore architecture and permeability, mechanical strength and viscoelasticity, degradation kinetics and transport behavior, as well as responsiveness to environmental cues such as pH, temperature, ionic strength, enzymes, or external stimuli. This tunability enables property customization and functional integration for diverse application needs. Owing to these designable and multifunctional features, hydrogels have been widely used in food, agriculture, cosmetics, and biomedicine. In biomedical research, representative applications include moist wound dressings and hemostatic materials, scaffolds for tissue engineering and regenerative medicine, wearable and implantable flexible devices, and drug delivery systems for local or targeted therapy [20,21,22].

For biomedical use and clinical translation, conventional hydrogels must satisfy both materials and biological requirements, such as controllable and reversible swelling or responsiveness, good processability and compatibility with sterilization, stable physicochemical performance, and reliable biocompatibility. However, many classic hydrogel systems rely on chemical crosslinkers, initiators, or polymerizable monomers during fabrication. When reactions are incomplete, or post-processing is insufficient, residual crosslinkers and unreacted monomers may remain, which raises potential biosafety concerns and can compromise in vivo safety and long-term stability. To address these limitations, a common strategy is to construct hybrid networks that combine natural polymers with synthetic polymers. This approach leverages the biocompatibility, degradability, and in some cases intrinsic bioactivity of natural polysaccharides or proteins, together with the structural tunability, mechanical adjustability, and functional integration offered by synthetic polymers, enabling refined control over network architecture, crosslink density, and responsive behavior. Within this framework, cellulose is an abundant and renewable natural polysaccharide supported by an established supply chain. It offers good biocompatibility and potential biodegradability, and its hydroxyl-rich chains provide reactive sites for chemical modification enabling the introduction of charges, dynamic linkages, or specific functional groups while maintaining a favorable safety profile, thereby addressing a broad range of biomedical needs [23]. Compared with other natural polymers such as silk protein [24], chitosan [25], sodium alginate [26], and hyaluronic acid [27], cellulose and its derivatives offer advantages in availability and cost control [28]. In addition, nanocellulose reinforcement and composite or double network designs can provide a more balanced combination of mechanical properties and shape retention, which supports scalable manufacturing and long-term stable biomedical applications. For example, Patel et al. prepared CNCs from spent coffee grounds (SCG) and incorporated it into a sodium alginate/CMC composite hydrogel. The added CNCs improved mechanical strength and structural stability, and it also influenced swelling and diffusion pathways by contributing to network formation and restricting segment mobility, leading to a smoother and more controllable release profile [29]. Liang et al. incorporated rigid CNFs and pH-responsive chitosan into a polyethylene glycol/sodium acrylate (PEG/SA) matrix and developed a mechanically robust and stimuli-responsive hydrogel, by combining dynamic metal–carboxylate coordination with polysaccharide-based reinforcement. In this system, CNFs form hydrogen bonds with the matrix through abundant hydroxyl groups, serving as physical junctions and limiting chain mobility. As a result, CNFs more effectively suppress excessive swelling and help preserve network integrity [30].

The construction of cellulose hydrogels is also typically based on physical crosslinking and chemical crosslinking [31,32]. Beyond these two major routes, several extended strategies have been developed. Examples include biologically triggered dynamic crosslinking that uses dynamic covalent bonds or specific interactions [33], photo-initiated crosslinking that enables rapid gelation with spatiotemporal control [34], and polymerization-based approaches in which monomer polymerization or copolymerization occurs together with crosslink formation [18]. For instance, dynamically crosslinked cellulose-based hydrogels are attractive for injectable delivery because they can combine shear-thinning behavior with structural recovery after administration [22]. Photo-initiated systems are useful when rapid gelation and spatiotemporal control are required, whereas polymerization-based or composite-network strategies are often adopted to improve shape retention, mechanical robustness, and release stability [29,30,35]. Accordingly, the choice of preparation route directly affects hydrogel performance and usually involves tradeoffs among mechanical support, responsiveness, process controllability, and biosafety [36].

Among stimuli-responsive cellulose hydrogels, pH-responsive systems have attracted particular attention because physiological and pathological pH differences are widely encountered in drug delivery, making pH one of the most practical endogenous triggers for release regulation. Tsubota et al. used aldehyde functionalized TEMPO-oxidized CNF (A-TOCNF) as a reactive platform, introduced glycol chitosan (GC) to form a dynamic imine network, and further leveraged the amino groups of doxorubicin (DOX) to achieve reversible covalent immobilization, thereby constructing a pH-responsive CNF hydrogel delivery system. By comparing DOX (with amino groups) and α-MS (without amino groups), the authors showed that covalent involvement markedly altered the release mechanism. In PBS (pH 7), DOX release remained very low and could be as low as approximately 10.2%, whereas α-MS release reached 54.0%, highlighting the contrast between diffusion-dominated release and covalent binding-mediated retention. The release profiles were further dictated by acid conditions and pH. DOX release stayed low at pH 7, for example, around 18.6%, while strong acidity at pH 2 promoted imine hydrolysis and increased release to about 68.8%. The authors also noted that overly strong acidic conditions could weaken covalent immobilization because of the hydrolysis equilibrium, which in turn affected both loading and release [37]. When interpreting such pH-responsive release behavior, it should be noted that, in charged cellulose hydrogels, the effective pH experienced by the network and the loaded drug may differ from the bulk medium because of Donnan partitioning and fixed-charge effects. As a result, the apparent pH-triggered release threshold depends not only on the external pH, but also on fixed-charge density and on whether drug retention is governed mainly by diffusion or by reversible covalent immobilization.

In addition to single pH-responsive designs, multi-stimulus platforms that combine pH responsiveness with other triggers have also been developed. Gao et al. reported a cellulose-based hydrogel delivery system with dual responsiveness to pH and oxidative stress. Using CMC as the backbone, the authors introduced dynamic boronate ester motifs, which made the network more prone to bond cleavage and structural relaxation under acidic conditions and thereby accelerated drug release. In their experiments, cytarabine showed a cumulative release of 61.3% at pH 5.0 after about 11 h, while the value decreased to 44.8% at pH 6.8 and to 34.6% at pH 7.4, indicating clear acid-triggered acceleration. The system was also responsive to hydrogen peroxide. When the H_2_O_2_ level was increased at pH 5.0, release was further accelerated, and the cumulative release increased to 84.6% under the condition described as 200 μmol. By using tumor-relevant acidity and elevated ROS as concurrent triggers, this design aims to keep the hydrogel more stable under normal tissue conditions while facilitating network opening and improved delivery efficiency at diseased sites, enabling controlled drug release [38]. Compared with purely swelling-dominated systems, this case shows how introducing dynamic bond chemistry into a charged cellulose network can shift release toward bond-cleavage-assisted triggering.

Gong et al. developed a cellulose hydrogel with dual responsiveness to pH and temperature. The main framework was constructed from β-cyclodextrin (β-CD) and CMC. Polyvinyl alcohol (PVA) was incorporated to form the first network, and a stable framework was obtained through citric acid-mediated esterification crosslinking. N-isopropyl acrylamide (NIPAM) monomer was then polymerized via photo initiation to build a second network for temperature-dependent regulation. In addition, cellulose-based carbon dots (C-CDs) prepared from lychee waste were introduced as crosslinking nodes, endowing the hydrogel with fluorescence tracking capability while maintaining a low toxicity crosslinking approach. In this system, pH responsiveness was provided by ionization of carboxyl groups in CMC, while temperature responsiveness was governed by the lower critical solution temperature (LCST) behavior of the poly (Nisopropylacrylamide) (PNIPAM). The dual network architecture enhanced swelling and water retention and improved mechanical stability. To address the limited loading of hydrophobic drugs, the authors combined host–guest inclusion within the hydrophobic cavity of β-CD with a denser network structure, achieving efficient ibuprofen loading and a smoother release profile, with a maximum encapsulation efficiency of 89%. The release curves were tunable across different pH and temperature conditions, and the cumulative release reached about 96% at pH 7.4 and 37 °C. In vitro tests indicated no marked cytotoxicity toward HepG2 cells, with cell viability remaining above 80%. The hydrogel also showed strong antibacterial activity against Escherichia coli and Staphylococcus aureus, with an inhibition rate exceeding 99% [35]. This case illustrates that, beyond ionization-driven pH sensitivity, the magnitude and smoothness of release are further shaped by network architecture and host–guest interactions. Taken together, these studies suggest that pH-responsive release from cellulose hydrogels is governed mainly by three coupled factors: network charge density, degree of substitution or functionalization, and crosslink architecture. First, the density of ionizable groups, such as carboxyl groups introduced through CMC or oxidation-derived cellulose, determines the extent of electrostatic repulsion and Donnan-type swelling under different pH conditions, and therefore strongly affects the apparent triggering threshold for release. Second, the degree of substitution or functionalization controls not only the number of ionizable or reactive groups available, but also their local accessibility and balance with hydrophobic or host–guest interactions, which in turn influence both drug retention and pH sensitivity. Third, crosslink architecture—including dynamic covalent linkages, ionic coordination, and double-network designs—governs network relaxation, diffusional resistance, and structural stability, thereby determining whether release is sharp, delayed, or sustained. Accordingly, pH-responsive behavior in cellulose hydrogels should not be interpreted solely in terms of the external pH, but rather in terms of how these structural variables collectively regulate ionization, swelling, and transport within the network.

Beyond pH-related designs, cellulose hydrogels have also been developed as platforms responsive to external physical triggers, which enable release to be activated in a more programmable manner. Gangurde et al. used a TEMPO-oxidized CNF hydrogel as a matrix and embedded thermosensitive liposomes loaded with indocyanine green into the network to produce a photosensitive cellulose hydrogel. Electrostatic interactions and the fibrillar network helped retain the liposomes locally, enabling long-term stability at 37 °C. Passive release of cationic liposomes was about 7% over 90 days. Release was then triggered using 808 nm near-infrared irradiation (NIR). Heat generated by indocyanine green increased local temperature, raised liposomal membrane permeability, and promoted rapid drug leakage. The release magnitude followed the irradiation dose, with about 50% release at 20 J/cm^2^ and about 80% release at 80 J/cm^2^. The response amplitude was also tunable via gel thickness, as thinner gels facilitated light penetration and more complete triggering. This design illustrates the feasibility of combining a cellulose hydrogel matrix with liposomal cargo to achieve NIR-triggered local release [39]. More broadly, it suggests that externally triggered release depends not only on the presence of a photosensitive component, but also on structural factors such as liposome retention within the fibrillar network, optical penetration through the hydrogel, and local heat transfer. As a result, the responsiveness of such systems is highly design-dependent and may vary substantially with network thickness and carrier organization.

Chumpitaz et al. prepared a cellulose-based hydrogel from cotton fabric waste and introduced superparamagnetic Fe_3_O_4_ nanoparticles to obtain magnetic responsiveness, while comparing two network routes, citric acid chemical crosslinking and freeze–thaw physical crosslinking. The materials exhibited superparamagnetism with a saturation magnetization of about 42 emu/g. Under an alternating magnetic field (187 kHz, 11.46 kA/m), a pronounced temperature increase was observed, ranging from about 8.3 to 24.5 °C. In water, the increase was about 8.3 to 9.3 °C, whereas in air it reached 20.8 to 24.5 °C within 5 min. These results provide a basis for magnetic hyperthermia and thermally triggered delivery concepts. After Fe_3_O_4_ incorporation, the compressive modulus of chemically crosslinked samples increased and could reach 152.4 ± 18.6 kPa, indicating that both crosslink architecture and nanoparticle incorporation substantially influenced mechanical performance. Although drug loading and release were not directly evaluated, the magnetic response and heating capability establish a material foundation for later magnetic field-triggered delivery and release [40]. Table 1 summarizes the representative cellulose-based hydrogels discussed in this section, together with their key delivery features and main limitations.

### 3.2. Cellulose-Based Aerogels

Aerogels are ultralight porous solids in which the pore space is largely filled with air. Unlike hydrogels, where a liquid phase occupies the network, aerogels are obtained by replacing the liquid component with air while retaining the three-dimensional porous network [41]. In drug delivery, their importance lies not simply in rehydration after administration, but in this dry-state porous architecture, which supports drug loading, maintenance of drugs in amorphous or highly dispersed states, and subsequent rehydration-governed release. Aerogels are further characterized by high porosity, large specific surface area, very low density, low thermal conductivity, low dielectric constant, and good biocompatibility [42]. These attributes have enabled broad applications of aerogels in thermal insulation, energy storage, oil and water separation, carbon dioxide capture, catalysis, and biomedicine [43].

Aerogel materials have evolved from early silica aerogels to polymer aerogels and, more recently, to bio-based aerogels, reflecting a gradual shift toward more environmentally benign materials. Bio-based aerogels include cellulose aerogels, chitosan aerogels, alginate aerogels, pectin aerogels, and protein aerogels [44]. Among these, cellulose and its derivatives offer three main advantages as aerogel precursors. First, cellulose is widely available and is one of the most abundant natural polymers, with renewability, sustainability, biodegradability, low toxicity, and environmental compatibility. Second, cellulose aerogels can be prepared through relatively simple processes with good reproducibility and potential for scale-up. Because cellulose chains contain abundant hydroxyl groups, additional crosslinkers are often unnecessary. Physical crosslinking can arise from hydrogen bonding within and between cellulose chains, enabling the self-assembly of a stable three-dimensional network. Third, cellulose has tunable surface functionality, which facilitates integration with a wide range of organic and inorganic components through chemical modification or physical blending to construct novel composite cellulose aerogels. These functional groups can also promote strong physical interactions with other materials. Together, these features make cellulose an important platform for developing hybrid aerogels with synergistically improved properties [45,46,47,48].

The preparation of cellulose aerogels typically involves three steps. First, cellulose or its derivatives are dissolved or dispersed, with the primary aim of weakening or disrupting the intra- and intermolecular hydrogen bonding network. Next, a sol–gel transition converts the system from a sol into a gel, where physical or chemical crosslinking generates a stable three-dimensional porous network. Finally, the solvent is removed while preserving the microstructure as much as possible [42]. Common approaches include supercritical drying, freeze drying, and conventional evaporative drying, and the drying route plays a critical role in determining pore architecture, specific surface area, and mechanical integrity. Supercritical CO_2_ drying is often favored because it reduces shrinkage and collapse associated with capillary forces at the liquid–gas interface, which is typically beneficial for producing cellulose aerogels with nanoporous structures and higher specific surface area [49].

Cellulose aerogels feature highly porous and continuous networks and offer favorable biocompatibility, biodegradability, and low toxicity, which can support high drug loading, improve the stability and dissolution performance of poorly soluble drugs, and facilitate sustained or controlled release designs. Drug incorporation commonly relies on physical confinement within the pore network and noncovalent interactions between the cellulose framework and drug molecules, such as hydrogen bonding and hydrophobic interactions. As a result, drugs are often present in amorphous or highly dispersed forms, which is advantageous for improving the dissolution of poorly soluble compounds [50]. Drug release, in turn, is closely linked to aerogel swelling in aqueous media, network stability, and pore connectivity. Cellulose source and morphology, crosslinking mode, and composite components can all markedly influence swelling kinetics and structural stability, leading to release behaviors dominated by diffusion, by swelling, or by a combination of both mechanisms, and thereby affecting release rates [51,52]. Valo et al. used beclomethasone dipropionate (BDP) as a model drug to evaluate source-dependent release behavior. When the aerogel framework was microcrystalline cellulose or red pepper-derived cellulose (RC), BDP exhibited a burst release of about 60% within the first 5 min, and the overall profile was similar to that of freeze-dried nanoparticles without a cellulose framework. In contrast, aerogels based on bacterial cellulose (BC), quince seed cellulose (QC), or TEMPO oxidized birch cellulose (TC) released only about 10 to 40% (BC) or about 10% (QC and TC) within the first 10 min, and release could extend to 700 min. These results indicate that celluloses from different sources may be chemically similar, yet they can lead to markedly different release profiles when used as aerogel matrices. Depending on the cellulose source, the release curve may be rapid or may become sustained [53].

Although pure cellulose aerogels already offer advantages such as low density, high porosity, and good biocompatibility, they also face intrinsic limitations in structural stability, mechanical performance, and functional diversity, which can restrict their broader use in complex pharmaceutical applications. Rahmanian et al. noted that pure cellulose aerogels often suffer from insufficient mechanical strength, limited thermal stability, and limited functionality, making it challenging to simultaneously satisfy requirements for structural integrity, controllable release, and multifunctional integration in drug delivery systems [54]. Accordingly, incorporating an organic or inorganic second phase to construct cellulose-based hybrid aerogels has become a practical route to improve performance and broaden the application scope. Cellulose can be combined with various components, including silica, graphene, graphene oxide, chitosan, metal oxides, and MOFs, to produce functional cellulose-based hybrid aerogels [49,54,55]. Such hybridization can markedly enhance mechanical properties and structural stability while preserving the advantages of a three-dimensional porous cellulose framework. It can also introduce additional functional elements through interfacial interactions and interpenetrating network structures, enabling coordinated tuning of drug loading, release behavior, and environmental responsiveness. In this context, cellulose hybrid aerogels are increasingly viewed as a key direction that bridges structural materials design with advanced pharmaceutical functional requirements.

Li et al. used carboxylated nanocellulose as the primary framework and developed a cellulose-based hybrid aerogel in which PEG and β-CD acted together to regulate performance for drug delivery. This work illustrates a hybrid strategy that integrates cellulose aerogels with functional molecular components. By jointly designing pore structure and molecular hosting sites, the system achieved high drug loading and more sustained release in gastric conditions [56]. Wu et al. prepared a pectin (PC) and CNF composite aerogel and showed that CNF-containing aerogels exhibited larger pores, thinner pore walls, and improved tensile and compressive properties. The release rate of active substances from the composite aerogel was also markedly reduced, indicating that CNF incorporation not only strengthened the material mechanically but also improved drug release behavior [57]. Xu et al. reported a similar trend in a collagen and cellulose hybrid aerogel (COL/CNF@TA). CNF promoted collagen self-assembly within a nanofibrillar network, forming an interpenetrating dual network porous framework, which substantially enhanced compressive performance under wet conditions. After adding tannic acid (TA), the pore structure became denser and more uniform. Using 5-fluorouracil (5-FU) as a model drug, the pure collagen aerogel showed rapid release, reaching 95.3% at about 2 h, whereas the CNF-containing composite aerogel suppressed rapid diffusion, extended release, and reduced burst release, reaching 81.4% at about 4 h. With the additional effect of TA, the drug-loaded aerogel displayed a more clearly staged sustained release behavior, and the release profile could be well fitted by a first-order model (R^2^ > 0.97) [58].

Regarding stimuli responsiveness, compared with hydrogels, where pH responsiveness is often expressed through bulk swelling of a fully hydrated network, pH-responsive behavior in cellulose aerogels is more strongly conditioned by rehydration kinetics, pore accessibility, and pH-dependent changes in network relaxation and diffusion pathways. Yue et al. prepared CNF bearing ionizable groups by TEMPO oxidation followed by amination. The functionalized CNF was then combined with collagen, and a layered, directionally aligned hybrid aerogel was obtained by coupling a collagen self-assembly pretreatment with directional freeze drying. Using 5-FU as a model drug, the authors found that introducing nonfunctionalized CNF into collagen could extend the time to reach the release plateau from about 2 h for pure collagen to about 4 h, while showing little dependence on pH. In contrast, introducing ionizable carboxyl or amino groups led to pronounced pH-dependent release. The collagen and carboxylated CNF aerogel showed the most evident sustained release at pH 3.0, reaching the release plateau at about 8 h with a cumulative release of 84.1%. The collagen and aminated CNF aerogel exhibited slower release under alkaline conditions, reaching the release plateau at about 6 h at pH 11.0 with a cumulative release of 90.7%. These results indicate that rational design of cellulose chemistry or network architecture enables fine-tuning of release behavior. The authors attributed the pH dependence to changes in electrostatic interactions between collagen and functionalized cellulose, which modulated network contraction or expansion and thereby altered diffusion pathways for 5-FU [59].

Liu et al. also used 5-FU as a model drug and developed a dual-responsive aerogel for drug delivery based on a CMC precursor, namely CMC/Ca^2+^/PNIPAM aerogels. CMC formed a stable pH-responsive network through Ca^2+^ chelation crosslinking and provided carboxyl sites that could undergo protonation and deprotonation. Interpenetration with a thermoresponsive poly (Nisopropylacrylamide) (PNIPAM) network produced an open porous structure with a porosity above 90%. The system responded to both pH and temperature. At 25 °C and pH 7.4, the cumulative release from CMC/PNIPAM and CMC/Ca^2+^/PNIPAM was about 56% and 52%, respectively, whereas at pH 3.0 it increased markedly to about 90% and 94%. Raising the temperature accelerated the overall release. At 37 °C and pH 7.4, the cumulative release increased to about 76% for CMC/PNIPAM and about 68% for CMC/Ca^2+^/PNIPAM. On this basis, the authors suggested that protonatable carboxyl groups in CMC enabled pH-triggered network contraction and expansion, while temperature-dependent conformational transitions of PNIPAM provided an additional route for thermal regulation. Together, these effects allowed combined release control characterized by pH selectivity and temperature-accelerated release within the same aerogel system. Overall, these studies highlight the progression of cellulose-based hybrid aerogels from passive porous matrices toward delivery platforms with tunable and stimuli-responsive behavior [60]. Table 2 summarizes the representative cellulose-based aerogels discussed in this section, including their major delivery functions and potential challenges.

### 3.3. Cellulose-Based Films

Films are flexible sheet-like substrates that can serve as local delivery platforms. A continuous polymer network within the film retains bioactive agents, which are then released in a sustained manner at the application site through hydration, swelling, and diffusion. This enables drug administration that is closer to spatiotemporal control of local concentration. In biomedical applications, films are widely used as drug delivery carriers and wound dressings, and they can also function as structural elements in tissue engineering systems to provide coverage, isolation, mechanical support, and microenvironmental regulation [61,62]. Application requirements dictate the key performance attributes. Films must be sufficiently compliant to conform to curved tissues, while maintaining adequate mechanical strength to preserve shape under wet conditions and tolerate friction. They also require appropriate adhesion and extensibility to maintain continuous contact at the target site and confine release to a defined region. In wound healing, films not only act as protective barriers but can also support repair by maintaining a moist environment and delivering antibacterial or anti-inflammatory agents.

Cellulose and its derivatives are commonly used as matrices for film-based systems because of their good biocompatibility, low toxicity, and film-forming capability. Within this review, cellulose-based films are discussed mainly as continuous film-type drug-delivery matrices for local retention, diffusional regulation, or directional release. The high density of hydroxyl groups along cellulose chains promotes a robust intermolecular interaction network, which can increase structural compactness and mechanical performance and improve water uptake, moisture retention, and morphological uniformity. Some cellulose-based films can also provide partial biodegradability and antibacterial functionality and have shown potential to promote re-epithelialization and tissue regeneration in wound dressing applications. The preparation of cellulose films typically involves two key steps: film formation and functionalization. Film formation can be achieved by solvent casting, spin coating, dip coating, or electrospraying to produce uniform, compact films. Layer-by-layer assembly and three-dimensional printing can further enable multilayer designs and precise thickness control. Functionalization is then performed to introduce drugs and antibacterial components through physical blending, adsorption followed by impregnation, or chemical crosslinking, with subsequent tuning of release kinetics [63,64,65,66,67]. For drug incorporation and release, cellulose films typically rely on their network structure to impose diffusional resistance and tortuous pathways. Film compactness, hydration state, and functional layering together determine the release profile. Multilayer strategies further expand this tunability. For example, introducing a hydrophobic backing layer outside the drug-loaded layer can reduce fluid ingress and unintended drug loss, while directing release primarily toward the tissue side to improve local delivery efficiency [68].

Building on these controlled release mechanisms, oral mucosal delivery has become an active area for cellulose film-based drug delivery. A number of products have already reached clinical use and the market, including FDA-approved buccal film formulations such as Onsolis, Zuplenz, Suboxone, Bunavail, Sympazan, Exservan, and Kynmobi [69]. These films are easy to administer and, once applied, are less likely to be expelled, which makes them particularly suitable for children, older adults, and patients with dysphagia. They can also reduce first-pass metabolism, improve bioavailability, and enable local delivery or controlled release. To meet clinical needs, the cellulose type and viscosity grade can be tuned. Variations in substituent chemistry and molecular weight lead to distinct rheological, mechanical, and hydration behaviors, allowing key attributes such as disintegration time, mucoadhesive strength, drug loading capacity, and release kinetics to be tailored. For instance, hydrophilic cellulose derivatives with lower viscosity are often preferred for fast-dissolving oral films, which rapidly hydrate in saliva and dissolve or disintegrate to achieve rapid drug release. In contrast, more hydrophobic and higher viscosity cellulose derivatives are better suited for buccal mucoadhesive films and for backing layers. After hydration, they more readily form a gel-like barrier that lengthens diffusion pathways and prolongs release. A backing layer can further limit saliva ingress into the drug-loaded layer, reduce drug loss, and promote unidirectional release [70,71,72,73].

Within this class of films, material selection governs hydration and swelling, while formulation ratios further define pore architecture and mechanical properties, which ultimately translate into the release profile. Accordingly, some studies have treated the composition ratio of cellulose derivatives as a key design variable to systematically optimize film structure and release kinetics. In a study initiated by Iqbal and Saeed, films containing different ratios of HPMC and CMC were compared. Adjusting the formulation markedly altered surface morphology and the microporous structure, and a higher fraction of CMC amplified pore features and modified swelling behavior. Release experiments indicated that film composition directly shaped the release pattern. Increasing the proportion of HPMC tended to accelerate levofloxacin release, whereas increasing the proportion of CMC favored sustained release of levofloxacin [74].

The studies discussed above mainly relied on formulation tuning within single-layer films to balance mechanical performance and controlled release. In contrast, another study used a multilayer architecture to create functional separation, assigning mucoadhesion, drug loading, and release directionality to different layers to improve robustness under oral conditions. Stie et al. employed distinct cellulose derivatives to differentiate the roles of a backing layer and a drug-containing layer, effectively treating saliva washout and drug dilution as controllable design factors. They proposed a multilayer cellulose film platform for peptide delivery across the oral mucosa. An HPMC freeze-dried foam was first prepared as an intermediate drug reservoir, loading the peptide desmopressin and providing sufficient dose capacity while maintaining overall flexibility and practical handling. A chitosan-based nanofiber layer was then electrospun onto the foam surface to strengthen buccal adhesion, resulting in a marked increase in work of adhesion to more than threefold compared with the foam alone. Finally, an EC backing film was spray-coated on the opposite side. Its hydrophobic nature limited saliva ingress and enabled unidirectional release. Release tests showed that the foam alone released about 80% within 30 min, whereas after introducing both the backing film and the nanofiber layer, the multilayer construct remained structurally intact for 3 h and achieved unidirectional release on the side protected by the backing. The nanofiber layer also served as a thin diffusional barrier, further slowing release. Importantly, in ex vivo permeation studies using porcine buccal mucosa, permeated drug from nanofiber-on-foam-on-film (NFF) was detectable after 1 h, reaching a cumulative amount of about 40 ng after 5 h, which corresponded to about 0.4% of the initial dose. In contrast, the commercial formulation MiniRin remained below the limit of quantification at all time points, suggesting that NFF may provide improved delivery efficiency for peptides across buccal mucosa [68].

Buccal films illustrate a key advantage of cellulose films for mucosal delivery, namely, hydration-driven residence combined with functional layering to support controlled release. Similar design principles can also be extended to other mucosal or epithelial interfaces exposed to highly dynamic fluids, such as the ocular surface. Habibullah et al. applied cellulose films for ocular delivery to replace frequent eye drop administration by increasing residence time. HPMC served as the film-forming matrix, NC were incorporated to reinforce the film network, and carboxymethylated polysaccharides were added to further tune hydration and adhesion. Moxifloxacin-loaded composite films were prepared by solvent casting, and different carboxymethylated polysaccharide formulations were compared systematically. The results indicated that the combined use of NC and carboxymethylated polysaccharides delayed corneal drug permeation and enhanced antibacterial activity against common Gram-positive and Gram-negative bacteria. Among the tested formulations, the carboxymethylated tamarind gum formulation produced a more pronounced inhibition zone. Cell-based assays supported biocompatibility. Moreover, an animal inflammation model suggested that the film could alleviate ocular inflammatory responses within a relatively short time frame [75]. Representative examples of cellulose-based films are summarized in Table 3.

### 3.4. Cellulose-Based Particulate and Dispersed Systems

Hydrogels, aerogels, and films are typically macroscopic, formable carriers. They regulate diffusion through 3D networks or compact film layers, thereby enabling drug loading and controlled release. In addition to these formats, drug delivery research frequently employs carriers at the micro- to nanoscale. Such systems are built around structural elements such as droplet interfaces, particulate shells, or lipid bilayers. In general, a stable dispersion is first established, active compounds are then positioned within the oil phase, the particle interior, or the lipid membrane, and release is subsequently governed by interfacial stability and permeability. Representative examples include Pickering emulsions, nanoparticles, microcapsules, core–shell particles, and cellulose-reinforced liposomes. These systems address well-defined needs in drug delivery. Many hydrophobic small molecules exhibit low solubility and are susceptible to photodegradation or oxidation. Particulate and dispersed carriers can sequester these actives in an oil phase, a hydrophobic core, or a lipid membrane, while an interfacial layer shields them from adverse conditions. This strategy can improve storage stability while maintaining fluidity or processability, and it can enhance delivery efficiency during the digestion process. Stimuli-responsive elements can also be incorporated into the interface or shell to enable condition-triggered release [10,76,77,78,79].

Within these carrier formats, cellulose and its derivatives typically play three roles. The first is interfacial stabilization. NC can adsorb at oil–water interfaces and form a particulate layer that suppresses droplet coalescence and improves emulsion stability. The second is the formation of shell or wall materials. EC and CMC can form a continuous matrix or a compact outer shell, enabling the fabrication of microcapsules and core–shell particles and allowing control over shell permeability. The third is surface coating and steric stabilization. Cellulose derivatives or NC can adsorb onto lipid vesicle surfaces, increase the viscosity of the continuous phase, and reduce vesicle aggregation. Notably, these roles are often coupled rather than exclusive. Many systems rely on interfacial adsorption together with electrostatic interactions and hydrogen bonding networks. A key advantage of cellulose is its rich and tunable surface chemistry, along with adjustable morphology and charge, which support both structural construction and fine regulation of interfacial energy and permeability [80,81,82,83].

#### 3.4.1. Pickering Emulsions

Pickering emulsions represent a typical interface-governed delivery system. The high aspect ratio of NC, together with its surface charge and partial amphiphilicity, makes it well-suited as a solid-particle stabilizer for Pickering emulsions [76]. Once adsorbed at the oil–water interface, the particles can form a robust interfacial layer that suppresses droplet coalescence and reduces the likelihood of emulsion breakdown under external perturbations. The properties of Pickering emulsions depend strongly on nanocellulose morphology and surface chemistry. Tuning surface charge and hydrophilic–hydrophobic balance regulates interfacial adsorption, while differences in nanocellulose crystalline structure can alter interfacial assembly and thereby affect emulsion stability [84,85,86,87].

**Table 3:** Representative cellulose-based films and particulate/dispersed systems in drug delivery.

Carrier Formats	Composition	Active Pharmaceutical Ingredient	Therapeutic Potential & Key Results	Potential Defects or Challenges	Reference
Films	HPMC; EC	Desmopressin	Improved mucoadhesion; Unidirectional release achieved; Better ex vivo permeation than comparator	Multilayer fabrication is more complex; Permeated fraction remained low; Translation still needs further validation	[68]
Films	HPMC; CMC	Levofloxacin	Composition-tunable release; Swelling and pore structure adjusted by formulation; Sustained release favored by higher CMC	Strongly formulation-dependent; Composition window may be narrow; Mechanical and release balance must be optimized	[74]
Films	HPMC; NC	Moxifloxacin	Delayed corneal permeation; Enhanced antibacterial activity; Good biocompatibility and anti-inflammatory potential	Long-term ocular safety not fully established; Clinical usability remains to be shown; Route-specific validation still needed	[75]
Pickering emulsions	CNC	Sesamolin	Improved sesamolin delivery; Selective cytotoxicity toward HCT116 cells; ROS-associated necrotic cell death observed	Administration route not established; Evidence mainly preclinical; Broader therapeutic validation is lacking	[88]

Rosalina et al. used carboxylated cellulose nanocrystals (cCNC) as the particulate emulsifier to construct an oil-in-water (O/W) Pickering emulsion for the delivery of the lipophilic active sesamolin. In vitro, cCNC itself showed low cytotoxicity toward HCT116 colon cancer cells and Vero nontumor cells, whereas the drug-loaded emulsion produced dose-dependent inhibition of HCT116 over a broader usable concentration range and showed a comparatively smaller effect on Vero cells [88]. Beyond storage protection, Pickering emulsions can also exhibit distinctive behavior during digestion. The interfacial particle layer may slow lipase access to lipid droplets and thus modify lipid hydrolysis kinetics. After entering the small intestine, bile salts and free fatty acids form mixed micelles, which often promote partitioning of hydrophobic actives into absorbable phases and increase bioaccessibility. Stimuli-responsive designs have also been explored, where interfacial particles aggregate or dissociate under defined conditions, leading to changes in emulsion stability and, at the desired stage, structural disruption that triggers release [89].

#### 3.4.2. Other Particulate and Dispersed Systems

Beyond Pickering emulsions, cellulose-based particulate and dispersed systems also include nanoparticles, microcapsules, core–shell particles, and cellulose-reinforced liposomes, which mainly rely on colloidal stabilization, shell-mediated retention, or vesicle protection.

Nanoparticles are particulate carriers that emphasize co-assembly and nanoscale protection. A common approach is to form composite nanoparticles from proteins and polysaccharides, allowing hydrophobic actives to partition into hydrophobic domains or become trapped within the network. CMC is widely used in such systems because its carboxylate groups increase hydrophilicity and provide sites for electrostatic interactions. This enables tuning of particle surface potential and colloidal stability, and stimuli responsiveness can be introduced when needed. After nanoparticles form, size and structural uniformity often determine storage stability and the reproducibility of release profiles. Processing variables, including the order of addition, shear intensity, and salt concentration, can alter particle structure and dispersion state and thereby influence release behavior [82,90,91,92].

Microcapsules and core–shell particles emphasize shell-mediated control. Microcapsules are typically in the micrometer range and encapsulate payloads within an outer shell, with release governed by shell permeability [93]. Core–shell particles further exploit layered architectures, where payloads can be placed in the core or distributed within the shell, facilitating multi-payload delivery and staged release [94]. Cellulose-reinforced liposomes belong to vesicular carriers. Liposomes can encapsulate both hydrophilic and hydrophobic compounds, but they often suffer from limited stability, and changes in ionic strength, as well as bile salt interactions, can accelerate vesicle restructuring [95]. Adsorption of cellulose derivatives or nanocellulose onto liposome surfaces can increase continuous phase viscosity and provide steric stabilization, thereby reducing aggregation and rupture [96]. Representative examples of cellulose-based particulate and dispersed systems are also listed in Table 3. Each cellulose-based carrier format has its own suitability range. Selection and customization should consider drug physicochemical properties and stability requirements, the intended therapeutic target, and the physiological environment at the site of action, to achieve reliable loading, controlled release, and improved therapeutic performance.

## 4. Application in Drug Delivery

### 4.1. Oral Drug Delivery

Oral administration is convenient, noninvasive, and well-suited for long-term therapy, and it is therefore often associated with high patient adherence. However, in the complex gastrointestinal tract, drug transit and residence time vary markedly with interindividual differences, fed or fasted state, and pH fluctuations, which makes it difficult to precisely control the sites of release and absorption. For drugs that irritate mucosa or exhibit local toxicity, oral dosing may also cause gastrointestinal discomfort and even mucosal injury, thereby constraining dose escalation and narrowing the therapeutic window [97]. Against this backdrop, cellulose and its derivatives-based oral delivery systems have attracted sustained interest in oral formulation design. CMC, cellulose acetate-based derivatives, EC, and nanocellulose recur in these systems because their ionizable groups, barrier effects, and porous structural roles can be exploited for gastric protection, intestinal triggering, prolonged gastric residence, and more site-selective release [98,99].

Pronounced pH gradients along the human gastrointestinal tract have stimulated the development of pH-responsive cellulose-based delivery systems, among which cellulose hydrogels are frequently used carriers [100]. In the acidic stomach environment, cellulose hydrogels can remain relatively stable and effectively encapsulate and protect entrapped drugs. Upon entry into the intestine, higher pH conditions trigger hydrogel swelling and promote drug release, enabling absorption across the intestinal mucosa and therapeutic action. Accordingly, for drugs that are unstable under acidic conditions, pH-responsive cellulose hydrogels can serve as effective oral delivery vehicles. Ibuprofen is a widely used nonsteroidal anti-inflammatory drug for arthritis, pain, and fever, but it can irritate the gastric mucosa, and long-term high-dose use may cause mucosal injury, ulcers, or bleeding. Sattari et al. used BNC as a backbone and incorporated CMC to construct 3D porous BNC/CMC hydrogel beads. Structural characterization showed that increasing CMC content in the culture medium increased bead crystallinity, reduced porosity, and produced a denser network. Functionally, pure BNC showed limited pH sensitivity, whereas CMC incorporation markedly enhanced pH responsiveness. In simulated gastric fluid (SGF, pH 1.2), CMC carboxyl groups were expected to remain largely nonionized, and bead swelling remained low. The apparent pKa of CMC has been reported to be around 4.3 under dilute aqueous conditions, although its ionization behavior may vary with electrolyte environment, degree of dissociation, and local chain context. In simulated intestinal fluid (SIF, pH 6.8), carboxyl ionization generated negative charges and inter-fiber electrostatic repulsion, leading to a pronounced increase in swelling. When ibuprofen was loaded into this system, higher CMC content strengthened pH responsiveness, slowed release in SGF, and accelerated release in SIF, which is expected to reduce ibuprofen exposure in the gastric phase and thereby mitigate gastric irritation. This work indicates that introducing CMC can preserve the structural stability and biocompatibility of BC while imparting tunable pH-triggered delivery behavior [101].

Similarly, Ahmadi et al. developed CMC/MIL-53 composite hydrogel beads that showed clear pH-dependent release using DOX as a model drug. Under simulated gastric conditions (pH 1.2), release was suppressed to about 27%. Under small intestinal pH (6.8), release was very low at about 3%, whereas under colon-relevant pH (7.4), it increased to about 70%, demonstrating a colon-targeted, controlled release profile. In this system, the CMC network provided an ingestible, bead-form matrix with ionic crosslinking stability and served as a structural platform for metal–organic frameworks (MOFs) formation and drug incorporation. Incorporation of MOF enhanced structural retention under acidic conditions and reduced premature leakage, which may lower the risk associated with DOX exposure in the upper gastrointestinal tract while enabling a release profile that better matches therapeutic needs in distal regions. Overall, this approach achieved site-selective delivery characterized by gastric phase protection and accelerated release at higher pH [102].

Akram et al. addressed key limitations of oral capecitabine for colorectal cancer chemotherapy, including premature release in the upper gastrointestinal tract, frequent dosing, and systemic toxicity, by developing a hydrogel system designed for colon site release. An enteric cellulose acetate phthalate-based crosslinked network was formed with pectin, guar gum, and acrylic acid, yielding porous gel carriers. Eleven formulations were screened under simulated gastric fluid and simulated intestinal fluid conditions. The optimized formulation F5 showed negligible swelling under acidic conditions and a suppressed release rate, providing effective protection in the gastric phase. Under pH 7.4 conditions, the network expanded markedly and enabled sustained release over 24 h, with cumulative release approaching or exceeding 80%. Acute oral toxicity was further evaluated in mice using the OECD 423 guideline, and no notable abnormalities were observed in hematological indices, biochemical parameters, or histology of major organs [103].

Importantly, intestinal release is not desirable for all drugs. Some compounds exhibit higher solubility in acidic media or present an absorption window in the upper gastrointestinal tract, where enhanced gastric dissolution together with controlled release can be advantageous. Imatinib is one such example, as it dissolves more readily under acidic conditions and shows limited dissolution under neutral to alkaline conditions, making gastric release and absorption more favorable [104]. Li and colleagues developed a nanocellulose composite aerogel aimed at selective drug release under gastric conditions, using imatinib as a model drug. Carboxylated nanocellulose served as a 3D framework, PEG was introduced to tune pore architecture, an β-CD was added to provide additional drug hosting and interaction sites, enabling high porosity together with increased drug loading capacity. Release was evaluated at pH 2.5, and compression of the aerogel into a tablet form was used to further prolong release. After compression, the maximum cumulative release was about 88%, and the release duration increased from 300 min to 660 min, and could be extended to about 11 h [56]. In other words, pH responsiveness in oral delivery is not a single design outcome but a context-dependent strategy. Its actual function is shaped by the interplay between formulation architecture, crosslink stability, and the drug’s gastrointestinal solubility behavior.

Nawaz et al. reported a gastric retentive floating microparticle formulation based on EC to improve the oral delivery of furosemide. Encapsulation efficiency was about 78 to 85%. In vitro tests in simulated gastric fluid showed buoyancy for more than 12 h and up to 24 h. The release profile featured a mild initial burst, with about 20 to 25% released in the first hour, followed by sustained release. Some formulations reached about 90% release at 12 h, whereas others showed about 83% cumulative release at 24 h, indicating prolonged drug availability. Mechanistically, ethyl cellulose contributed a hydrophobic diffusion barrier and structural stabilization of the microparticles, supporting extended gastric residence and sustained release, which aligns well with drugs that benefit from an upper gastrointestinal absorption window [105].

The objectives of oral platforms are not limited to delivering active drugs. For certain conditions, it can be more important to capture and remove harmful species within the gastrointestinal tract, thereby reducing systemic burden and improving clinical outcomes. Wilson’s disease provides a representative example. Wilson’s disease, also known as hepatolenticular degeneration, is an autosomal recessive disorder caused by mutations in the ATP7B gene. Impaired biliary excretion of excess copper leads to abnormal copper accumulation in the liver, brain, cornea, and other tissues, resulting in progressive hepatic injury and neuropsychiatric manifestations [106,107,108]. Cellulose is attractive for oral adsorbent or chelating materials because it is generally biocompatible, widely available, and minimally absorbed from the gastrointestinal tract, which supports local intestinal action [109,110].

Ding et al. prepared a cellulose-based oral copper chelation material and compared how different modification strategies affected copper adsorption. Using MCC as the substrate, surface modification introduced amino and thiol binding sites. In vitro adsorption tests showed that unmodified MCC exhibited limited copper uptake, with an adsorption of about 13.45% and a capacity of 3.36 mg/g. PEI-modified MCC (MCC-PEI) increased adsorption to 62.08% with a capacity of 15.52 mg/g. Further incorporation of SiO_2_ nanoparticles together with thiol-rich functionalities yielded MCC-NS, which reached 85.52% and 21.38 mg/g. For comparison, orally administered activated carbon showed about 14.83% and 3.71 mg/g. Overall, the modified MCC materials outperformed the control, demonstrating that the low gastrointestinal absorption of cellulose combined with surface functionalization can enhance copper capture and enable local gastrointestinal action [111]. Nevertheless, these characteristics do not by themselves establish safety, and such systems still require careful evaluation before clinical translation. Table 4 summarizes the representative oral delivery applications discussed in this section, together with their therapeutic potential and remaining challenges.

### 4.2. Transdermal Drug Delivery

Compared with many other routes, transdermal drug delivery offers practical advantages, including the possibility of self-administration, relatively stable release rates, and sustained dosing. If discomfort occurs, therapy can be terminated rapidly by removing the device. Transdermal delivery can also partially bypass hepatic first-pass metabolism and reduce the impact of gastrointestinal conditions, which may improve bioavailability for certain drugs and decrease gastrointestinal adverse effects. Nevertheless, the major limitation remains the skin barrier, particularly the stratum corneum, which restricts the types of molecules and the deliverable dose. In addition, skin irritation potential, adhesive stability, and comfort during prolonged application must be carefully balanced in formulation design. In this context, HPMC-, EC-, and CMC-based systems, together with nanocellulose-reinforced designs, have been widely used in transdermal and microneedle patches because they offer good biocompatibility, stable adhesion, and sustained release [112,113].

For local therapy, the advantages of transdermal delivery are particularly evident. Antibacterial agents or active constituents from traditional Chinese medicine can be delivered to deeper skin layers or localized lesions, increasing local exposure while reducing systemic risk associated with broader distribution. Zhang et al. developed a sulfonated bacterial cellulose and chitosan (SBC/CS) composite hydrogel for transdermal delivery in psoriasis. Sulfonation introduced sulfonic acid groups into bacterial cellulose, which provided ROS scavenging antioxidant activity to mitigate the inflammatory microenvironment and also strengthened interactions with methotrexate (MTX), improving drug loading and enabling better control over release. Incorporation of chitosan formed a hydrogen-bonded network that imparted self-healing and tissue adhesion, supporting skin attachment and a stable administration interface. In transdermal evaluations, the hydrogel showed MTX release and permeation behavior that was more suitable for skin application, and it achieved efficacy comparable to a clinical control in a psoriasis animal model while maintaining good skin compatibility [114]. Gao et al. used a one-pot method to incorporate Yunnan Baiyao extract and borneol into a polymer network and introduced cellulose nanofibers to reinforce the framework. Notoginsenoside R1 was released continuously over 72 h with a cumulative release of about 88%. In Franz diffusion experiments using porcine skin, the patch showed stable transdermal flux, with a cumulative permeation of about 45% at 72 h and about 7% residual drug remaining within the skin. The patch exhibited high stretchability and could adhere gently to skin, reducing the risk of irritation. It also showed low temperature tolerance, with a freezing point down to about 27.7 °C. Cytocompatibility and skin safety were verified, and an inhibition zone of about 8 mm was observed. Overall, this work combined nanofiber reinforced mechanics with the local therapeutic goals of traditional Chinese medicine components, addressing both comfort and controlled release while also incorporating use scenario factors such as cold resistance into the design [115].

Transdermal delivery is not limited to local therapy, and systemic therapy represents another important application scenario. Such applications impose higher demands on drug loading capacity, sustained release, and effective flux across the skin barrier. Pandya et al. used sodium CMC (CMC-Na) as the primary matrix and incorporated an ionic liquid MOF composite, [TMG][Ol]@UiO-66-NH_2_, to construct a hybrid hydrogel for 5-FU delivery. Supported by the CMC-Na matrix, the hydrogel exhibited overall properties suitable for patch-based administration and achieved high 5-FU loading. For two hybrid hydrogels with different composite ratios, G1 (0.1:1) and G2 (0.25:1), the loading capacities were 671 mM and 397.8 mM, respectively. In vitro skin permeation studies showed a 48 h cumulative permeation of 76.4% for G1 and 82.7% for G2, demonstrating the feasibility of using cellulose derivatives as hydrogel matrices for transdermal delivery of poorly soluble drugs [116].

Among strategies to enhance transdermal delivery efficiency, microneedle patches are particularly notable because they can pierce the stratum corneum with minimal invasiveness and markedly shorten diffusion pathways. Microneedle systems typically need to satisfy two sets of requirements, namely sufficient drug loading with controllable release, and adequate mechanical strength with safe use during insertion. Cellulose-based materials can act as drug carrier matrices and can also be engineered into device components, such as functional layers or structural elements, thereby broadening their utility in microneedle-enabled transdermal delivery.

One approach is to fabricate cellulose into porous paper or fibrous felt sheets that function as dry drug reservoirs or controlled release layers within microneedle patches. In this design, the drug reservoir is separated from the insertion unit. After insertion, the microneedles absorb interstitial fluid and swell to form transport pathways, allowing the drug to be continuously supplied from the reservoir into the skin, which supports controlled release and adjustable dosing. Abraham et al. proposed a combined system described as SmartReservoirs (SRs) coupled with hydrogel-forming microneedles (HF-MNs). A hydrophilic model drug, amitriptyline hydrochloride (AMT), was first loaded into a cellulose-based porous paper matrix to create an independent drug reservoir, which was then paired with drug-free HF-MNs. After the microneedles penetrated the stratum corneum, they absorbed interstitial fluid and swelled. AMT migrated from the SRs into the swollen hydrogel pathways and then diffused continuously into the skin, enabling controlled and delayed transdermal delivery. Two cellulose paper matrices were compared. Photocopy paper SR-P showed nearly twice the drug loading of tissue paper SR-T and produced higher levels of skin deposition and permeation over 24 h, indicating that the type of cellulose paper used for the reservoir can strongly influence loading as well as permeation and deposition distributions. This suggests that dosing can be tuned by adjusting microstructural features of the paper reservoir. Notably, AMT in the SRs existed both as an amorphous phase within pores and as a partially crystalline phase on the surface. The amorphous fraction dominated early release, while the crystalline fraction, limited by dissolution, contributed to later sustained release. Together, these forms produced a gradually increasing release profile [117].

Another line of research integrates drug loading and skin penetration more directly by constructing the microneedle device itself from cellulose-based materials. Tseng et al. demonstrated this concept using cellulose aerogel microneedles. Compared with conventional dense polymer microneedles, cellulose aerogel microneedles retain a highly interconnected porous network, which can markedly increase drug loading capacity. Through appropriate structural design, the microneedles also maintain sufficient mechanical strength to pierce the stratum corneum and can release the drug effectively after insertion [118].

Zhang and Wang developed a CMC-based hydrogel-forming microneedle (HMN) platform, using citric acid as a mild chemical crosslinker to generate a stable three-dimensional network while maintaining biocompatibility. By tuning the extent of citric acid crosslinking, the microneedles achieved the mechanical strength required for skin insertion. Drug loading was straightforward, and after application, the microneedles rapidly absorbed water and swelled, enabling rapid drug release. Insulin was used as the model drug, and controlled release relied on an “adsorb and flip” strategy. At pH 2.0, insulin is positively charged and can adsorb electrostatically onto abundant carboxyl groups within the CMC network crosslinked by citric acid, leading to high loading. After insertion into the skin, the physiological pH in the dermis shifts the charge state of insulin and induces electrostatic repulsion, triggering rapid release. At pH 7.4, about 74% was released within 5 min, and release was nearly complete within 15 min, whereas at pH 2.0, only about 2.6% was released over 2 h. These results suggest that cellulose-based HMNs can couple mild loading conditions with pH-triggered rapid release, offering a materials strategy for transdermal delivery of protein therapeutics with attention to both safety and manufacturability [119].

To support combination therapy, microneedle patches can also co-deliver two drugs. Yousaf et al. addressed the slow onset of action and limited oral adherence in acute migraine treatment by incorporating sumatriptan succinate and naproxen sodium into dissolving microneedle patches. The goal was to use the transdermal route to bypass first-pass metabolism while enabling both rapid onset and sustained dosing. PVA and HPMC were used as the needle matrix, and a 100-needle array was fabricated by cast molding. Each needle had a height of about 500 μm and a base diameter of about 200 μm, with well-formed tips and good uniformity. Mechanical testing indicated practical insertion performance, with an insertion force of about 5.28 N, and finger pressure was sufficient to pierce the surface barrier without frequent breakage. In insertion tests, needles reached depths of about 250 to 380 μm, indicating effective traversal of the stratum corneum. X-Ray diffraction (XRD) suggested that both active pharmaceutical ingredients were crystalline in their raw forms, but became more amorphously dispersed within the microneedle matrix, which can facilitate dissolution and diffusion. The release profile showed an initial faster phase followed by sustained release. Within the first hour, sumatriptan release was about 20% and naproxen release was about 10%. Cumulative release reached about 87% and 80% at 24 h, respectively. Under the same in vitro conditions, commercial oral tablets released about 24%, highlighting the microneedle system’s higher release efficiency and improved control over release patterns. In vivo skin irritation assessment showed no obvious erythema or edema, supporting preliminary tolerability for transdermal use [120].

Release kinetics can also be differentiated for different drugs through compartmentalized designs. Bahmani et al. developed a composite hydrogel microneedle patch using sodium CMC. Dissolving microneedles loaded with lidocaine hydrochloride were first formed by cast molding. Gelatin and sodium CMC microcapsules containing the same drug were then incorporated into the backing layer, so that the microneedles provided rapid analgesia while the microcapsules enabled sustained release to prolong action, producing a biphasic release profile. The microcapsules had a size of about 50 to 264 μm, an encapsulation efficiency of about 75.8%, and a drug loading of about 9.8%. Release from the microcapsules was more pronounced under acidic conditions. The in vitro release curve showed two distinct phases, with rapid release during the first 10 min reaching about 71.5%, followed by a sustained release phase that continued up to 240 min. The authors also verified microneedle mechanical properties and insertion capability, and reported antibacterial performance and good cytocompatibility, with cell viability remaining above 86% [121]. Table 5 summarizes the representative transdermal delivery applications discussed in this section, including patch- and microneedle-based systems.

### 4.3. Localized and Targeted Delivery

As previously mentioned, cellulose derivatives and nanocellulose can support localized and targeted delivery through their structure–property–function relationships, including mucoadhesion, pH responsiveness, injectable network formation, and surface functionalization capacity. Such strategies can offer two main benefits. First, they may reduce systemic adverse effects, which is particularly important for patients with compromised immunity or complex concomitant medications. Second, they can establish higher and more sustained drug concentrations at the target site, which may improve therapeutic efficiency and extend the duration of action. In practice, delivery strategies for targeting can be grouped into three categories. The first is anatomical localization, where drugs are placed directly at or near the lesion, such as within periodontal pockets, joint cavities, tumor interstitium, or postoperative cavities. The second is microenvironment-triggered targeting, where carriers respond to local cues, including the mildly acidic conditions common in inflamed or tumor tissues, altered enzyme profiles, or elevated oxidative stress. The third is molecular recognition-based targeting, where receptor-mediated uptake is exploited, for example via folate receptors or CD44, or where lesion-associated surface markers are used to promote cellular internalization [78,122,123]. This section focuses on injectable localized delivery and ligand-mediated targeted systems that achieve site-specific retention and enhanced local efficacy.

Ghosh et al. developed an injectable, pH-responsive hydrogel for local delivery. Oxidized CMC provided aldehyde groups and was directly mixed with a modified chitosan solution at physiological temperature. Simple mixing at 37 °C led to rapid gelation within 60 s. The material formed a dynamic network through Schiff base linkages and electrostatic interactions, giving it shear-thinning behavior and rapid structural recovery after cessation of shear. This combination supports minimally invasive injection and retention at the target site. Biosafety evaluation indicated good cytocompatibility and hemocompatibility, together with antioxidant activity and antibacterial potential. Using diclofenac sodium as a model drug, the hydrogel showed a biphasic release profile at pH 6.8, with an initial faster phase followed by a slower phase, reaching about 80% cumulative release at 24 h. Release was notably slower than that of a polysaccharide-only control formulation. Overall, this study suggests that cellulose-based injectable systems can be tailored to lesion-specific pH conditions to support targeted local therapy [124].

Oral disease therapy is often challenged by short residence time because the oral cavity is highly dynamic. Han et al. developed an injectable short fiber dual network hydrogel for local administration into periodontal pockets to treat periodontitis. TEMPO oxidized bacterial nanocellulose (OBNC) was further grafted with chitosan to obtain OBNC-CH short fibers, which were mixed with a GelMA prepolymer and then photo-crosslinked to form a dual network structure. The resulting hydrogel could be injected to fill irregular periodontal pockets and remained structurally stable under repeated mechanical loading in the oral environment, showing strong fatigue resistance. By loading metronidazole, the hydrogel inhibited periodontal pathogens and, in a rat periodontitis model, reduced alveolar bone resorption while promoting bone regeneration and tissue repair. Overall, the nanocellulose short fiber reinforced design enabled a balance between mechanical robustness and localized antibacterial drug delivery in the dynamic oral setting [125].

In tumor therapy, many studies have focused on developing responsive formulations that exploit features of the tumor microenvironment. Local injection represents another practical strategy. By administering drugs directly at the tumor site, injection-based therapy may reduce the need for invasive procedures and can be more acceptable for patients. This local approach can lower systemic toxicity, limit damage to healthy tissues, and support sustained dosing. Accordingly, injectable materials must provide reliable injectability to enable accurate administration [126].

Zhou et al. developed a cellulose hydrogel-based composite delivery system for gastric cancer that can be administered via endoscopic injection, where injectability is a critical requirement. Thiolated CMC (CMC-SH) was used to prepare microgels that co-carried hydrophilic 5-FU and hydrophobic curcumin. These drug-loaded microgels were then dispersed within a hydrogel matrix formed from dialdehyde cellulose (DAC) and gelatin, yielding the composite 5-FU+Cur/CMC-SH m@DACG. In injection tests, the composite could be smoothly extruded through a syringe, and rheological measurements showed typical shear thinning behavior, supporting both ease of injection and shape stability after administration. Dynamic imine linkages within the network endowed the hydrogel with self-healing capability, allowing it to conform to irregular defect geometries and rapidly recover after mechanical disruption, thereby maintaining structural integrity and protecting the drug-loaded microgels. The system also enabled controlled release over distinct time scales, with rapid 5-FU release during the first 4 h (about 95%) and sustained curcumin release over 108 h, approaching complete release. In vitro, the drug-free formulation showed good biocompatibility toward normal gastric epithelial cells, whereas the dual drug-loaded system produced stronger growth inhibition and apoptosis induction in two gastric cancer cell lines. Overall, this work highlights the multiple roles that cellulose derivatives can play in injectable delivery materials and provides a materials and delivery design framework for minimally invasive local combination therapy in gastric cancer [127].

In another study, Chatap et al. extracted CNFs from wheat fibers, introduced amino groups using 3-aminopropyltriethoxysilane (APTES), loaded curcumin, and then covalently grafted folic acid onto the fiber surface via N-(3-dimethylaminopropyl)-N’-ethylcarbodiimide hydrochloride (EDC) and N-hydroxysuccinimide (NHS) coupling to construct a folate-targeted nanofiber system for lung cancer delivery. Leveraging folate receptor-related recognition, the authors aimed to enhance tumor cell uptake and observed dose-dependent inhibition in A549 lung cancer cells, with an IC_50_ of about 100 µM/mL. The material showed pH-dependent release, with cumulative release of about 85% at pH 6.8 and about 64% at pH 7.4, indicating faster release under mildly acidic conditions. The formulation was further processed into a dry powder inhaler and evaluated using a cascade impactor, yielding an emitted dose of 42.7% and a fine particle fraction of 15.3%. These results suggest manufacturing feasibility and a certain potential for pulmonary deposition. From a targeting perspective, combining ligand recognition with an inhalation route may further support lung-focused delivery [128]. Table 6 summarizes the representative localized and targeted delivery applications discussed in this section, together with their key results and potential limitations.

## 5. Conclusions

This review summarizes the materials foundation, structural tunability, and carrier construction strategies of cellulose and its derivatives for drug delivery. We systematically discuss representative cellulose-based platforms, including hydrogels, aerogels, and films, with a focus on design rationales, drug loading approaches, and principles governing release control. By establishing unified structure–property–function relationships for each cellulose derivative, we clarify the translation of material properties across carrier formats and administration routes, and further highlight representative advances across oral delivery, transdermal delivery, and local administration. Overall, cellulose and its derivatives constitute a materials family that combines sustainability with strong potential for rational structural design. Through molecular-level substitution and nanostructural tuning, these materials can be optimized in a coordinated manner across various properties, including swelling behavior, pore architecture, interfacial interactions, and mechanical stability. At the carrier level, cellulose hydrogel design commonly couples network architecture with swelling and diffusion processes, and integrates dynamic linkages or stimuli-responsive mechanisms to enable synergistic control over release. Cellulose aerogels, due to their high porosity and large specific surface area, are particularly effective in dispersing poorly soluble drugs and supporting sustained release. Cellulose films are well-suited for adhesive residence, barrier functions, and directional release, and they have shown clear potential for translation toward product development. At the application level, cellulose-based systems have demonstrated recognizable clinical relevance for site-specific oral release, sustained transdermal dosing, and localized therapy. Despite substantial progress, stronger and more comprehensive evidence is still required for clinical translation of cellulose-based drug delivery systems.

First, manufacturability will be a key barrier when cellulose-based systems transition from laboratory studies to industrial production. Many laboratory formulations depend on specific solvent systems, modification or crosslinking conditions, and drying processes to achieve targeted pore structures and mechanical properties. Upon scale-up, however, challenges such as increased energy demand, difficulty in solvent recovery, and complex processing steps often emerge, resulting in limited batch consistency and poorly controlled costs. For clinical translation, it is advisable to define, at an early stage, a reproducible and controllable processing window and to prioritize greener, simplified, and robust preparation routes that reduce process sensitivity and improve batch consistency.

Second, safety should not be inferred solely from the general biocompatibility of the base material. Instead, it should be supported by a verifiable body of evidence for the safety of the complete delivery system. The favorable compatibility of cellulose does not automatically translate into the safety of the final formulation, because chemical modifications, composite components, crosslinkers, and residual species, nanoscale morphological differences, and degradation products can all alter host responses. At present, many studies still rely primarily on in vitro cellular data, while systematic in vivo evidence on toxicity, immunogenicity, and inflammatory responses remains limited. Future work should strengthen studies on long-term in vivo stability and degradation and establish safety evidence using models that better reflect clinically relevant exposure conditions.

Third, functional integration and stimulus-responsive design should evolve from qualitative responsiveness toward performance that is quantifiable, predictable, and calibratable. Current stimuli-responsive mechanisms are often limited in complexity, and response speed and sensitivity can be insufficient, leading to release profiles that deviate from expectations under real physiological conditions. Future designs should parameterize response thresholds, response rates, and release kinetics, and use approaches such as multi-stimulus coordination, structural compartmentalization, and role-differentiated release to improve robustness against environmental variability. In addition, to better align with real therapeutic needs, enabling co-delivery of multiple active agents and implementing programmable release schemes within a single platform will be an important direction.

Fourth, artificial intelligence and advanced manufacturing may substantially improve development efficiency for cellulose-based delivery systems. Machine learning can support rapid multi-objective optimization and, together with in-line characterization data, enable predictive models that shorten formulation development cycles and reduce trial and error. Meanwhile, three-dimensional printing offers a route to programmable fabrication of shape and structure for personalized dosing, gradient architectures, microneedle arrays, and composite patches. These opportunities also introduce stricter requirements for rheology, curing mechanisms, printing resolution, sterilization, and storage stability, and therefore need to be advanced in parallel with manufacturable standards.

Fifth, from a regulatory standpoint, evaluation of cellulose-based delivery systems extends beyond efficacy to include material source, purity and residual control, stability assessment, packaging and sterilization validation, and process control with batch consistency under a quality management system. It is advisable to clarify regulatory classification early based on the intended application. Using a quality by design framework, critical quality attributes and critical process parameters can be defined and standardized, and reproducible evaluation workflows can be used to continuously accumulate evidence suitable for regulatory submissions, thereby reducing the risk of extensive rework during late-stage scale-up and registration.

In summary, future competitiveness of cellulose-based delivery systems will depend less on novelty of materials alone and more on scalable manufacturing readiness, robust safety evidence, predictable release control under complex physiological conditions, and a quality system aligned with regulatory expectations. With continued progress in greener processes, AI-assisted formulation optimization, and standardized evaluation frameworks, cellulose-based materials are well positioned to deliver more clinically viable solutions for precise and effective therapy.

## Figures and Tables

**Table 1:** Representative cellulose-based hydrogels in drug delivery.

Carrier Formats	Composition	Active Pharmaceutical Ingredient	Therapeutic Potential & Key Results	Potential Defects or Challenges	Reference
Hydrogels	CNC; SA; CMC	Not specified *	Improved mechanical strength and structural stability; Restricted swelling and diffusion; Smoother and more controllable release	Drug specificity not emphasized; Route relevance unclear; Mainly structural demonstration	[29]
Hydrogels	CNF; chitosan	Not specified *	Suppressed excessive swelling; Preserved network integrity; Improved mechanical robustness	Limited therapeutic validation; Drug loading not central; Application scenario not clearly defined	[30]
Hydrogels	A-TOCNF	Doxorubicin	pH-sensitive controlled delivery; Reversible covalent immobilization; Acid-triggered release via imine hydrolysis	Trigger relied on strong acidity; Route-specific relevance limited; Conditions may be difficult to generalize	[37]
Hydrogels	CMC	Cytarabine	Tumor-microenvironment-responsive release; Acidic and oxidative acceleration; Improved delivery selectivity	In vivo validation still limited; Multi-stimulus control may be harder to calibrate; System complexity increased	[38]
Hydrogels	β-CD; CMC	Ibuprofen	High encapsulation efficiency; Tunable pH/temperature-responsive release; Good antibacterial and biocompatibility performance	Multi-component design is complex; Scale-up may be difficult; Release behavior depends on several coupled variables	[35]
Hydrogels	TOCNF	Indocyanine green	Very low passive release; NIR-triggered on-demand local release; Output tunable by irradiation dose and thickness	Sensitive to optical penetration; Heat transfer affects performance; Response depends strongly on structural design	[39]
Hydrogels	Cellulose from cotton waste	Not specified *	Magnetic responsiveness achieved; Heating capability demonstrated; Potential basis for dual-trigger delivery	Drug release not directly tested; Therapeutic performance not established; Still at material-foundation stage	[40]

* Not specified: Active pharmaceutical ingredient not explicitly reported in the original study.

**Table 2:** Representative cellulose-based aerogels in drug delivery.

Carrier Formats	Composition	Active Pharmaceutical Ingredient	Therapeutic Potential & Key Results	Potential Defects or Challenges	Reference
Aerogels	MCC/RC/BC/QC/TC	Beclomethasone dipropionate	Cellulose source shaped release profile; Some systems reduced burst release; Sustained release extended up to 700 min	Strong source dependence; Predictability may be limited; Matrix selection becomes critical	[53]
Aerogels	Carboxylated NC	Imatinib	High loading capacity; Gastric-condition-selective release; Prolonged release after compression	Hybrid composition adds complexity; Reproducibility may be challenging; Processing route may affect scale-up	[56]
Aerogels	CNF; pectin	Not specified *	Improved pore structure; Better tensile and compressive properties; Slower release rate	DDS function not fully route-defined; Active drug context limited; More matrix-focused than therapeutic	[57]
Aerogels	CNF; collagen; tannic acid	5-FU	Reduced burst release; Prolonged and staged sustained release; Improved wet compressive performance	Multi-component architecture is complex; Formulation control may be difficult; Translation evidence remains limited	[58]
Aerogels	Functionalized CNF; collagen	5-FU	Clear pH-dependent release; Tunable diffusion pathways; Functional chemistry regulated network behavior	Rehydration conditions influence response; Electrostatic environment is important; Release behavior may vary across systems	[59]
Aerogels	CMC	5-FU	Combined pH selectivity and thermal acceleration; High porosity retained; Dual-trigger release regulation achieved	Multi-stimulus control may reduce reproducibility; Response threshold needs calibration; Composite behavior is harder to predict	[60]

* Not specified: Active pharmaceutical ingredient not explicitly reported in the original study.

**Table 4:** Representative oral applications in drug delivery.

Route of Administration	Cellulose-Based Carrier Formats	Active Pharmaceutical Ingredient	Therapeutic Potential & Key Results	Potential Defects or Challenges	Reference
Oral	BNC/CMC-based hydrogels	Ibuprofen	pH-responsive oral delivery potential; Reduced gastric exposure; Enhanced intestinal release behavior	Sensitive to gastrointestinal conditions; Depends on network density and ionization; In vivo validation still limited	[101]
Oral	CMC-based hydrogels	Doxorubicin	Colon-targeted oral delivery; Suppressed upper-GI release; Accelerated release at pH 7.4	Colon selectivity mainly based on in vitro release; Broader biological validation needed; Composite preparation adds complexity	[102]
Oral	CAP-based hydrogels	Capecitabine	Colon-specific delivery design; Minimal swelling in gastric phase; Sustained release over 24 h at pH 7.4	Formulation is relatively complex; Long-term robustness not fully shown; Translation still uncertain	[103]
Oral	EC-based microparticles	Furosemide	Gastric-retentive delivery; Long buoyancy time; Sustained release in gastric medium	Performance may vary with GI motility; Fed/fasted state may affect retention; Route behavior needs broader validation	[105]
Oral	MCC-based oral chelation material	Copper ions	Gastrointestinal-restricted copper sequestration; Strongly improved adsorption after modification; Potential for Wilson’s disease management	Safety cannot be inferred directly; Clinical translation remains preliminary; Long-term GI effects need evaluation	[111]

**Table 5:** Representative transdermal applications in drug delivery.

Route of Administration	Cellulose-Based Carrier Formats	Active Pharmaceutical Ingredient	Therapeutic Potential & Key Results	Potential Defects or Challenges	Reference
Transdermal	SBC/CS composite hydrogel	Methotrexate	Precision psoriasis treatment potential; Improved loading and local skin compatibility; Good efficacy in animal model	Application remains disease-specific; Evidence still preclinical; Broader transdermal generalizability unclear	[114]
Transdermal	CNF-reinforced composite hydrogel patch	Yunnan Baiyao	Continuous release over 72 h; Balanced mechanics and skin comfort; Added anti-freezing functionality	Multi-component herbal system is harder to standardize; Mechanism is less defined; Translation may be formulation-sensitive	[115]
Transdermal	CMC-Na-based hydrogels	5-FU	High loading capacity; Strong 48 h skin permeation; Feasible transdermal delivery for poorly soluble drug	Composite complexity may raise safety concerns; Manufacturability needs assessment; Long-term skin compatibility needs more evidence	[116]
Transdermal	Cellulose paper-based SmartReservoir + HF-MNs	Amitriptyline hydrochloride	Controlled and delayed transdermal delivery; Dosing tunable by paper microstructure; Reservoir–microneedle separation strategy	Performance depends on paper type; Reservoir reproducibility is critical; System structure is relatively complex	[117]
Transdermal	CMC-based hydrogels	Insulin	Gentle loading and rapid release; pH-triggered transdermal insulin delivery; Nearly complete release within 15 min	Loading/storage conditions may need tight control; Strong pH dependence may limit robustness; Further in vivo translation needed	[119]
Transdermal	PVA/HPMC-based dissolving microneedle patch	Sumatriptan succinate; naproxen sodium	Dual-drug migraine therapy potential; Good insertion performance; Higher release efficiency than oral comparator	Dual-drug formulation increases complexity; Dose uniformity may be harder to control; Still requires broader translational validation	[120]
Transdermal	CMC-based hydrogels	Lidocaine hydrochloride	Biphasic release profile; Rapid analgesia plus sustained release; Good cytocompatibility and antibacterial performance	In vivo comparative evidence is limited; System design is relatively complex; Long-term patch performance not fully established	[121]

**Table 6:** Representative localized and targeted delivery applications in drug delivery.

Route of Administration	Cellulose-Based Carrier Formats	Active Pharmaceutical Ingredient	Therapeutic Potential & Key Results	Potential Defects or Challenges	Reference
Localized/lesion-adapted injection	CMC-based hydrogels	Diclofenac sodium	Lesion-adapted local delivery; Good cytocompatibility and hemocompatibility; Controlled release under pH 6.8	Application scope remains relatively narrow; Clinical scenario is localized only; Further therapeutic validation is needed	[124]
Localized oral administration	OBNC-CH short-fiber dual-network hydrogel	Metronidazole	Injectable periodontal-pocket therapy; Strong fatigue resistance in oral environment; Promoted tissue repair in vivo	Site-specific application limits generalizability; Administration may depend on procedure; Translation beyond oral lesions is limited	[125]
Localized/endoscopic injection	CMC-SH-based hydrogels	5-FU; curcumin	Minimally invasive local combination therapy; Shear-thinning and self-healing behavior; Dual-timescale co-release achieved	Multi-component injectable system is hard to standardize; Endoscopic use increases practical complexity; Scale-up may be challenging	[127]
Pulmonary/targeted	CNF-based dry powder inhaler	Curcumin	Folate-targeted lung delivery potential; pH-dependent release behavior; Inhalation-ready formulation with deposition potential	Targeting evidence remains mainly preclinical; Cell-level validation dominates; In vivo delivery efficiency still needs confirmation	[128]

## Data Availability

No new data were created or analyzed in this study. Data sharing is not applicable to this article.

## References

[1] Li X., Ji X., Chen K., Yuan X., Lei Z., Ullah M.W., Xiao J., Yang G. (2021). Preparation and Evaluation of Ion-Exchange Porous Polyvinyl Alcohol Microspheres as a Potential Drug Delivery Embolization System. Mater. Sci. Eng. C.

[2] Garg T., Arora S., Pahwa R. (2025). Cellulose and Its Derivatives: Structure, Modification, and Application in Controlled Drug Delivery. Futur. J. Pharm. Sci..

[3] Goh K.Y., Ching Y.C., Ng M.H., Chuah C.H., Julaihi S.B.J. (2022). Microfibrillated Cellulose-Reinforced Alginate Microbeads for Delivery of Palm-Based Vitamin E: Characterizations and in Vitro Evaluation. J. Drug Deliv. Sci. Technol..

[4] You C., Lin H., Ning L., Ma N., Wei W., Ji X., Wei S., Xu P., Zhang D., Wang F. (2024). Advances in the Design of Functional Cellulose Based Nanopesticide Delivery Systems. J. Agric. Food Chem..

[5] Govindarasu M., Palanisamy S., Joy J.G., Sharma G., You S., Kim J.-C. (2025). Advances of Nanocellulose and Cellulose-Based Derivatives for Biomedical Applications. Cellulose.

[6] Cheng W., Zhu Y., Jiang G., Cao K., Zeng S., Chen W., Zhao D., Yu H. (2023). Sustainable Cellulose and Its Derivatives for Promising Biomedical Applications. Prog. Mater. Sci..

[7] Das M., Lalsangi S., Santra S., Banerjee R. (2024). Nanocellulose as a Carrier for Improved Drug Delivery: Progresses and Innovation. J. Drug Deliv. Sci. Technol..

[8] Xue H., Zhu C., Wang Y., Gu Q., Shao Y., Jin A., Zhang X., Lei L., Li Y. (2025). Stimulus-Responsive Cellulose Hydrogels in Biomedical Applications and Challenges. Mater. Today Bio..

[9] Wang Y., Qi J., Zhang M., Xu T., Zheng C., Yuan Z., Si C. (2024). Cellulose-Based Aerogels, Films, and Fibers for Advanced Biomedical Applications. Chem. Eng. J..

[10] Xu Y., Guo J., Wei Z., Xue C. (2025). Cellulose-Based Delivery Systems for Bioactive Ingredients: A Review. Int. J. Biol. Macromol..

[11] Mehrabi A., Jalise S.Z., Hivechi A., Habibi S., Kebria M.M., Haramshahi M.A., Latifi N., Karimi A., Milan P.B. (2024). Evaluation of Inherent Properties of the Carboxymethyl Cellulose (CMC) for Potential Application in Tissue Engineering Focusing on Bone Regeneration. Polym. Adv. Techs.

[12] Layek B., Mandal S. (2020). Natural Polysaccharides for Controlled Delivery of Oral Therapeutics: A Recent Update. Carbohydr. Polym..

[13] Arca H.C., Mosquera-Giraldo L.I., Bi V., Xu D., Taylor L.S., Edgar K.J. (2018). Pharmaceutical Applications of Cellulose Ethers and Cellulose Ether Esters. Biomacromolecules.

[14] Qin Z., Ng W., Ede J., Shatkin J.A., Feng J., Udo T., Kong F. (2024). Nanocellulose and Its Modified Forms in the Food Industry: Applications, Safety, and Regulatory Perspectives. Compr. Rev. Food Sci. Food Saf..

[15] Seneviratne D.M., Whiteside E.J., Windus L.C., Burey P.P., Ward R., Annamalai P.K. (2025). Emerging Biomedical Applications of Sustainable Cellulose Nanocrystal-Incorporated Hydrogels: A Scoping Review. Gels.

[16] Foster E.J., Moon R.J., Agarwal U.P., Bortner M.J., Bras J., Camarero-Espinosa S., Chan K.J., Clift M.J.D., Cranston E.D., Eichhorn S.J. (2018). Current Characterization Methods for Cellulose Nanomaterials. Chem. Soc. Rev..

[17] Melro L., Alves C., Fernandes M., Rocha S., Mehravani B., Ribeiro A.I., Azevedo S., Cardoso V.F., Carvalho Ó., Dourado N. (2025). Bacterial Nanocellulose as a Versatile Scaffold for Biomedical Applications: Synthesis, Functionalization, and Future Prospects. Appl. Mater. Today.

[18] Bashir S., Hina M., Iqbal J., Rajpar A.H., Mujtaba M.A., Alghamdi N.A., Wageh S., Ramesh K., Ramesh S. (2020). Fundamental Concepts of Hydrogels: Synthesis, Properties, and Their Applications. Polymers.

[19] Chang L., Du H., Xu F., Xu C., Liu H. (2024). Hydrogel-Enabled Mechanically Active Wound Dressings. Trends Biotechnol..

[20] Nasra S., Patel M., Shukla H., Bhatt M., Kumar A. (2023). Functional Hydrogel-Based Wound Dressings: A Review on Biocompatibility and Therapeutic Efficacy. Life Sci..

[21] Li L., Cheng X., Huang Q., Cheng Y., Xiao J., Hu J. (2022). Sprayable Antibacterial Hydrogels by Simply Mixing of Aminoglycoside Antibiotics and Cellulose Nanocrystals for the Treatment of Infected Wounds. Adv. Healthc. Mater..

[22] Jiang X., Zeng F., Yang X., Jian C., Zhang L., Yu A., Lu A. (2022). Injectable Self-Healing Cellulose Hydrogel Based on Host-Guest Interactions and Acylhydrazone Bonds for Sustained Cancer Therapy. Acta Biomater..

[23] Long Y., Dimde M., Adler J.M., Vidal R.M., Povolotsky T.L., Nickl P., Achazi K., Trimpert J., Kaufer B.B., Haag R. (2024). Sulfated Cellulose Nanofiber Hydrogel with Mucus-Like Activities for Virus Inhibition. ACS Appl. Mater. Interfaces.

[24] Arndt T., Chatterjee U., Shilkova O., Francis J., Lundkvist J., Johansson D., Schmuck B., Greco G., Nordberg Å.E., Li Y. (2024). Tuneable Recombinant Spider Silk Protein Hydrogels for Drug Release and 3D Cell Culture. Adv. Funct. Mater..

[25] Xu J., Chang L., Xiong Y., Peng Q. (2024). Chitosan-Based Hydrogels as Antibacterial/Antioxidant/Anti-Inflammation Multifunctional Dressings for Chronic Wound Healing. Adv. Healthc. Mater..

[26] Yang X., Huang C., Wang H., Yang K., Huang M., Zhang W., Yu Q., Wang H., Zhang L., Zhao Y. (2024). Multifunctional Nanoparticle-Loaded Injectable Alginate Hydrogels with Deep Tumor Penetration for Enhanced Chemo-Immunotherapy of Cancer. ACS Nano.

[27] Burdick J.A., Prestwich G.D. (2011). Hyaluronic Acid Hydrogels for Biomedical Applications. Adv. Mater..

[28] Li B., Xu M., An B., Sun W., Teng R., Luo S., Ma C., Chen Z., Li J., Li W. (2025). Mechanical and Thermal Responsive Chiral Photonic Cellulose Hydrogels for Dynamic Anti-Counterfeiting and Optical Skin. Mater. Horiz..

[29] Patel D.K., Cha J., Won S.-Y., Han S.S. (2025). Upcycled Coffee Waste into Nanocellulose-Reinforced Alginate–Carboxymethyl Cellulose Hydrogels for Tunable Drug Delivery. Int. J. Biol. Macromol..

[30] Liang Y., Luo T., Li F., Wang J., Tang Y. (2025). Comparative Study of Injectable and Stimuli-Responsive Hydrogels Reinforced with Cellulose Nanofibers and Chitosan for Controlled Drug Delivery. Chem. Eng. J..

[31] Nasution H., Harahap H., Dalimunthe N.F., Ginting M.H.S., Jaafar M., Tan O.O.H., Aruan H.K., Herfananda A.L. (2022). Hydrogel and Effects of Crosslinking Agent on Cellulose-Based Hydrogels: A Review. Gels.

[32] Zhou J., Wang X., Li Y., Li H., Lu K. (2020). Preparation of Cellulose Nanocrystal-Dressed Fluorinated Polyacrylate Latex Particles via RAFT-Mediated Pickering Emulsion Polymerization and Application on Fabric Finishing. Cellulose.

[33] Cheng Y., Wang Y., Wang Y., Tan P.-C., Yu S., Li C., Li Z.-Y., Li Q.-F., Zhou S.-B., Wang C. (2025). Microenvironment-Feedback Regulated Hydrogels as Living Wound Healing Materials. Nat. Commun..

[34] Hua Y., Xia H., Jia L., Zhao J., Zhao D., Yan X., Zhang Y., Tang S., Zhou G., Zhu L. (2021). Ultrafast, Tough, and Adhesive Hydrogel Based on Hybrid Photocrosslinking for Articular Cartilage Repair in Water-Filled Arthroscopy. Sci. Adv..

[35] Gong J., Ching Y.C., Huang S., Niu Q.J., Li A., Yong C.K., Sampath Udeni Gunathilake T.M., Hai N.D., Hock C.C. (2025). A Dual Stimuli-Responsive Cellulose-Based Double Network Hydrogel Crosslinked with Fluorescent Carbon Dots for Controlled Drug Release. React. Funct. Polym..

[36] Di J., Li J., Sun C., Xu L., Li X. (2025). Advances in Cellulose-Based Hydrogels for Drug Delivery: Preparation, Modification and Challenges. Gels.

[37] Tsubota H., Park J., Kang H., Kang D., Miura Y., Saito T., Kono N., Kawasaki R., Jung S.H., Jung J.H. (2026). Aldehyde-Functionalized Cellulose Nanofiber Hydrogels for pH-Sensitive Drug Delivery via Dynamic Imine Bonding. ACS Omega.

[38] Gao J., Wang R., Liang Z., Zhao Z., Kong Y., Zhou M. (2025). Controlled Delivery of Cytarabine in Sodium Carboxymethyl Cellulose/Copper-Doped Prussian Blue Hydrogels for Chemotherapy and Chemodynamic Therapy. ACS Appl. Nano Mater..

[39] Gangurde P., Gounani Z., Zini J., Polez R.T., Österberg M., Lauren P., Lajunen T., Laaksonen T. (2025). Harnessing Liposomal Nanocellulose Hydrogel for NIR-Light Driven on-Demand Drug Delivery. Carbohydr. Polym. Technol. Appl..

[40] Chumpitaz D., Mariños Y., Peña E., Sencia J.J.H., Zapata A., Ponce S., Félix L.L., Quispe L.T., López R., Gutarra A. (2025). Sustainable Magnetic Hydrogels Derived from Cotton Fabric Waste as Candidates for Dual-Triggered Drug Release Applications. Mater. Today Commun..

[41] García-González C.A., Sosnik A., Kalmár J., De Marco I., Erkey C., Concheiro A., Alvarez-Lorenzo C. (2021). Aerogels in Drug Delivery: From Design to Application. J. Control. Release.

[42] Yan G., Chen B., Zeng X., Sun Y., Tang X., Lin L. (2020). Recent Advances on Sustainable Cellulosic Materials for Pharmaceutical Carrier Applications. Carbohydr. Polym..

[43] Dhua S., Gupta A.K., Mishra P. (2022). Aerogel: Functional Emerging Material for Potential Application in Food: A Review. Food Bioprocess. Technol..

[44] Chen B., Zheng Q., Zhu J., Li J., Cai Z., Chen L., Gong S. (2016). Mechanically Strong Fully Biobased Anisotropic Cellulose Aerogels. RSC Adv..

[45] Ahmadi M., Madadlou A., Saboury A.A. (2016). Whey Protein Aerogel as Blended with Cellulose Crystalline Particles or Loaded with Fish Oil. Food Chem..

[46] Seantier B., Bendahou D., Bendahou A., Grohens Y., Kaddami H. (2016). Multi-Scale Cellulose Based New Bio-Aerogel Composites with Thermal Super-Insulating and Tunable Mechanical Properties. Carbohydr. Polym..

[47] Long L.-Y., Weng Y.-X., Wang Y.-Z. (2018). Cellulose Aerogels: Synthesis, Applications, and Prospects. Polymers.

[48] Nguyen B.N., Cudjoe E., Douglas A., Scheiman D., McCorkle L., Meador M.A.B., Rowan S.J. (2016). Polyimide Cellulose Nanocrystal Composite Aerogels. Macromolecules.

[49] Liu Z., Zhang S., He B., Wang S., Kong F. (2021). Synthesis of Cellulose Aerogels as Promising Carriers for Drug Delivery: A Review. Cellulose.

[50] Selvaraj S., Chauhan A., Dutta V., Verma R., Rao S.K., Radhakrishnan A., Ghotekar S. (2024). A State-of-the-Art Review on Plant-Derived Cellulose-Based Green Hydrogels and Their Multifunctional Role in Advanced Biomedical Applications. Int. J. Biol. Macromol..

[51] Rostamitabar M., Ghahramani A., Seide G., Jockenhoevel S., Ghazanfari S. (2022). Drug Loaded Cellulose–Chitosan Aerogel Microfibers for Wound Dressing Applications. Cellulose.

[52] Ren J., Hasuo K., Wei Y., Tabata I., Hori T., Hirogaki K. (2023). Fabrication of Monolithic Para-Aramid Nanofibers/Cellulose Acetate Composite Aerogels with Homogeneous and Durable Cross-Linked Nanostructures for Filtration, Adsorption, and Drug Delivery. ACS Appl. Nano Mater..

[53] Valo H., Arola S., Laaksonen P., Torkkeli M., Peltonen L., Linder M.B., Serimaa R., Kuga S., Hirvonen J., Laaksonen T. (2013). Drug Release from Nanoparticles Embedded in Four Different Nanofibrillar Cellulose Aerogels. Eur. J. Pharm. Sci..

[54] Rahmanian V., Pirzada T., Wang S., Khan S.A. (2021). Cellulose-Based Hybrid Aerogels: Strategies toward Design and Functionality. Adv. Mater..

[55] Lei C., Gao J., Ren W., Xie Y., Abdalkarim S.Y.H., Wang S., Ni Q., Yao J. (2019). Fabrication of Metal-Organic Frameworks@cellulose Aerogels Composite Materials for Removal of Heavy Metal Ions in Water. Carbohydr. Polym..

[56] Li Y., Liu X., Liu Z., Wang S., Kong F. (2025). Fabrication of Controllable Structure of Nanocellulose Composite Aerogel for Targeted Drug Delivery. Carbohydr. Polym..

[57] Wu W., Wu Y., Lin Y., Shao P. (2022). Facile Fabrication of Multifunctional Citrus Pectin Aerogel Fortified with Cellulose Nanofiber as Controlled Packaging of Edible Fungi. Food Chem..

[58] Xu M., Yue C., Hu M., Liao M., Zhang R., Cai G., Cheng B. (2025). Tannic Acid-Mediated Multifunctional Collagen/Cellulose Nanofiber Composite Aerogels for Sustained Drug Release, Antibacterial Properties, and Hemostasis. ACS Appl. Polym. Mater..

[59] Yue C., Ding C., Hu M., Zhang R., Cheng B. (2024). Collagen/Functionalized Cellulose Nanofibril Composite Aerogels with pH-Responsive Characteristics for Drug Delivery System. Int. J. Biol. Macromol..

[60] Liu Z., Zhang S., Gao C., Meng X., Wang S., Kong F. (2022). Temperature/pH-Responsive Carboxymethyl Cellulose/Poly (N-Isopropyl Acrylamide) Interpenetrating Polymer Network Aerogels for Drug Delivery Systems. Polymers.

[61] Choi V., Rohn J.L., Stoodley P., Carugo D., Stride E. (2023). Drug Delivery Strategies for Antibiofilm Therapy. Nat. Rev. Microbiol..

[62] Priya S., Choudhari M., Tomar Y., Desai V.M., Innani S., Dubey S.K., Singhvi G. (2024). Exploring Polysaccharide-Based Bio-Adhesive Topical Film as a Potential Platform for Wound Dressing Application: A Review. Carbohydr. Polym..

[63] Chen L.-H., Doyle P.S. (2022). Thermogelling Hydroxypropyl Methylcellulose Nanoemulsions as Templates to Formulate Poorly Water-Soluble Drugs into Oral Thin Films Containing Drug Nanoparticles. Chem. Mater..

[64] Nawaz A., Latif M.S., Shah M.K.A., Elsayed T.M., Ahmad S., Khan H.A. (2023). Formulation and Characterization of Ethyl Cellulose-Based Patches Containing Curcumin-Chitosan Nanoparticles for the Possible Management of Inflammation via Skin Delivery. Gels.

[65] Carmona P., Poulsen J., Westergren J., Pingel T.N., Röding M., Lambrechts E., De Keersmaecker H., Braeckmans K., Särkkä A., Von Corswant C. (2023). Controlling the Structure of Spin-Coated Multilayer Ethylcellulose/Hydroxypropylcellulose Films for Drug Release. Int. J. Pharm..

[66] Dechojarassri D., Okada T., Tamura H., Furuike T. (2023). Evaluation of Cytotoxicity of Hyaluronic Acid/Chitosan/Bacterial Cellulose-Based Membrane. Materials.

[67] Schmidt L.M., dos Santos J., de Oliveira T.V., Funk N.L., Petzhold C.L., Benvenutti E.V., Deon M., Beck R.C.R. (2022). Drug-Loaded Mesoporous Silica on Carboxymethyl Cellulose Hydrogel: Development of Innovative 3D Printed Hydrophilic Films. Int. J. Pharm..

[68] Stie M.B., Öblom H., Hansen A.C.N., Jacobsen J., Chronakis I.S., Rantanen J., Nielsen H.M., Genina N. (2023). Mucoadhesive Chitosan- and Cellulose Derivative-Based Nanofiber-on-Foam-on-Film System for Non-Invasive Peptide Delivery. Carbohydr. Polym..

[69] Cheng H., Wang Y., Hong Y., Wu F., Shen L., Lin X. (2025). Characteristics, Preparation and Applicability in Oral Delivery Systems of Cellulose Ether-Based Buccal Films. Drug Deliv..

[70] Gupta M.S., Kumar T.P., Gowda D.V., Rosenholm J.M. (2021). Orodispersible Films: Conception to Quality by Design. Adv. Drug Deliv. Rev..

[71] Göbel A., Breitkreutz J. (2022). Concept of Orodispersible or Mucoadhesive “Tandem Films” and Their Pharmaceutical Realization. Pharmaceutics.

[72] Takeuchi Y., Kawamoto M., Tahara K., Takeuchi H. (2018). Design of a New Disintegration Test System for the Evaluation of Orally Disintegrating Films. Int. J. Pharm..

[73] Ansari M., Sadarani B., Majumdar A. (2018). Optimization and Evaluation of Mucoadhesive Buccal Films Loaded with Resveratrol. J. Drug Deliv. Sci. Technol..

[74] Iqbal A., Saeed R. (2025). Synthesis, Characterization, and Optimization of Cellulose-Based Hydrogel Films for Controlled Levofloxacin Release with Reversed-Phase High-Performance Liquid Chromatography Validation. J. Chin. Chem. Soc..

[75] Habibullah S., Meher J.R., Das M., Das T., Swain R., Mohanty B., Mallick S. (2025). Moxifloxacin in HPMC-Nanocellulose Composite Film for the Management of Ocular Inflammation: Effect of Carboxymethylated Gum on Permeation and Antimicrobial Activity. Int. J. Biol. Macromol..

[76] Ji C., Wang Y. (2023). Nanocellulose-Stabilized Pickering Emulsions: Fabrication, Stabilization, and Food Applications. Adv. Colloid. Interface Sci..

[77] Dezhman M., Sani M.Z., Ahmadi M.K.B., Rahdan F., Alizadeh E., Dianat-Moghadam H. (2025). Cellulose-Based Nanoparticles: Preparation Strategies and Biomedical Applications. Polym. Bull..

[78] Babaei-Ghazvini A., Patel R., Vafakish B., Yazdi A.F.A., Acharya B. (2024). Nanocellulose in Targeted Drug Delivery: A Review of Modifications and Synergistic Applications. Int. J. Biol. Macromol..

[79] Mohanta V., Madras G., Patil S. (2014). Layer-by-Layer Assembled Thin Films and Microcapsules of Nanocrystalline Cellulose for Hydrophobic Drug Delivery. ACS Appl. Mater. Interfaces.

[80] Li Z., Yu D. (2023). Controlled Ibuprofen Release from Pickering Emulsions Stabilized by pH-Responsive Cellulose-Based Nanofibrils. Int. J. Biol. Macromol..

[81] Li Y., Zhang H., Zhao Y., Lv H., Liu K. (2024). Encapsulation and Characterization of Proanthocyanidin Microcapsules by Sodium Alginate and Carboxymethyl Cellulose. Foods.

[82] Chang C., Wang T., Hu Q., Luo Y. (2017). Caseinate-Zein-Polysaccharide Complex Nanoparticles as Potential Oral Delivery Vehicles for Curcumin: Effect of Polysaccharide Type and Chemical Cross-Linking. Food Hydrocoll..

[83] Faroux J.M., Ureta M.M., Tymczyszyn E.E., Gómez-Zavaglia A. (2020). An Overview of Peroxidation Reactions Using Liposomes as Model Systems and Analytical Methods as Monitoring Tools. Colloids Surf. B Biointerfaces.

[84] Ni Y., Gu Q., Li J., Fan L. (2021). Modulating in Vitro Gastrointestinal Digestion of Nanocellulose-Stabilized Pickering Emulsions by Altering Cellulose Lengths. Food Hydrocoll..

[85] Saffarionpour S. (2020). Nanocellulose for Stabilization of Pickering Emulsions and Delivery of Nutraceuticals and Its Interfacial Adsorption Mechanism. Food Bioprocess. Technol..

[86] Li X., Li J., Gong J., Kuang Y., Mo L., Song T. (2018). Cellulose Nanocrystals (CNCs) with Different Crystalline Allomorph for Oil in Water Pickering Emulsions. Carbohydr. Polym..

[87] Kalashnikova I., Bizot H., Cathala B., Capron I. (2011). New Pickering Emulsions Stabilized by Bacterial Cellulose Nanocrystals. Langmuir.

[88] Rosalina R., Weerapreeyakul N., Sutthanut K., Kamwilaisak K., Sakonsinsiri C. (2025). Nanocellulose-Based Pickering Emulsion of Sesamolin Manifested Increased Anticancer Activity and Necrosis in Human Colon Cancer (HCT116) Cells. Int. J. Biol. Macromol..

[89] Li Y., Fei S., Yu D., Zhang L., Li J., Liu R., Tan M. (2022). Preparation and Evaluation of Undaria Pinnatifida Nanocellulose in Fabricating Pickering Emulsions for Protection of Astaxanthin. Foods.

[90] Caicedo Chacon W.D., Verruck S., Monteiro A.R., Valencia G.A. (2023). The Mechanism, Biopolymers and Active Compounds for the Production of Nanoparticles by Anti-Solvent Precipitation: A Review. Food Res. Int..

[91] Zhang L.-Q., Niu B., Yang S.-G., Huang H.-D., Zhong G.-J., Li Z.-M. (2016). Simultaneous Preparation and Dispersion of Regenerated Cellulose Nanoparticles Using a Facile Protocol of Dissolution–Gelation–Isolation–Melt Extrusion. ACS Sustain. Chem. Eng..

[92] Javanbakht S., Shaabani A. (2019). Carboxymethyl Cellulose-Based Oral Delivery Systems. Int. J. Biol. Macromol..

[93] Wani S.U.D., Ali M., Mehdi S., Masoodi M.H., Zargar M.I., Shakeel F. (2023). A Review on Chitosan and Alginate-Based Microcapsules: Mechanism and Applications in Drug Delivery Systems. Int. J. Biol. Macromol..

[94] Van Der Kooij R.S., Steendam R., Frijlink H.W., Hinrichs W.L.J. (2022). An Overview of the Production Methods for Core–Shell Microspheres for Parenteral Controlled Drug Delivery. Eur. J. Pharm. Biopharm..

[95] Varma K., Jude S., Nair R.V.R., Varghese B.A., Jacob J., Amalraj A., Kuttappan S. (2021). Novel Formulation of Liposomal Lutein Using Nanofiber Weaving (NFW) Technology: Antioxidant Property and in Vitro Release Studies. Food Hydrocoll. Health.

[96] Wang X., Liu L., Xia S., Muhoza B., Cai J., Zhang X., Duhoranimana E., Su J. (2019). Sodium Carboxymethyl Cellulose Modulates the Stability of Cinnamaldehyde-Loaded Liposomes at High Ionic Strength. Food Hydrocoll..

[97] Noreen S., Pervaiz F., Ashames A., Buabeid M., Fahelelbom K., Shoukat H., Maqbool I., Murtaza G. (2021). Optimization of Novel Naproxen-Loaded Chitosan/Carrageenan Nanocarrier-Based Gel for Topical Delivery: Ex Vivo, Histopathological, and In Vivo Evaluation. Pharmaceuticals.

[98] Awad A., Madla C.M., McCoubrey L.E., Ferraro F., Gavins F.K.H., Buanz A., Gaisford S., Orlu M., Siepmann F., Siepmann J. (2022). Clinical Translation of Advanced Colonic Drug Delivery Technologies. Adv. Drug Deliv. Rev..

[99] Stielow M., Witczyńska A., Kubryń N., Fijałkowski Ł., Nowaczyk J., Nowaczyk A. (2023). The Bioavailability of Drugs—The Current State of Knowledge. Molecules.

[100] Zong S., Wen H., Lv H., Li T., Tang R., Liu L., Jiang J., Wang S., Duan J. (2022). Intelligent Hydrogel with Both Redox and Thermo-Response Based on Cellulose Nanofiber for Controlled Drug Delivery. Carbohydr. Polym..

[101] Sattari A., Basirattalab A., Alemzadeh I. (2025). Fabrication of pH-Sensitive Bacterial Cellulose/Carboxymethyl Cellulose Hybrid Hydrogel Beads in Agitated Culture for Oral Drug Delivery. Can. J. Chem. Eng..

[102] Ahmadi M., Javanbakht S., Shaabani A., Kazeminava F. (2026). In-Situ Synthesis of Aluminum-Based Metal-Organic Framework within the Aluminum-Crosslinked Carboxymethyl Cellulose Hydrogel Beads: A Safe Carrier for Anticancer Oral Drug Delivery. J. Drug Deliv. Sci. Technol..

[103] Akram S., Malik N.S., Zeeshan M., Tulain U.R., Anwar M., Mahmood A., Javaid A., Hussain S., Jabeen A., Rahman A.U. (2025). Design, Development, and Characterization of Stimuli-Responsive Polymeric Carriers for Colon-Specific Drug Delivery: A Promising Approach for Capecitabine. J. Drug Deliv. Sci. Technol..

[104] Kamali M., Jafari H., Taati F., Mohammadnejad J., Daemi A. (2024). Synthesis of Chitosan Polyethylene Glycol Antibody Complex for Delivery of *Imatinib* in Lung Cancer Cell Lines. J. Biochem. Amp. Mol. Tox.

[105] Nawaz M., Rehman S., Shoukat H., Farooq M.I., Sarfraz M., Khan K.U., Khan S., Saqib K.A., Rai N., Mahmood H. (2026). Gastro-Retentive Floating Microparticles for Enhanced Oral Delivery of Furosemide: Formulation, Characterization, and Controlled Release. Powder Technol..

[106] Shribman S., Marjot T., Sharif A., Vimalesvaran S., Ala A., Alexander G., Dhawan A., Dooley J., Gillett G.T., Kelly D. (2022). Investigation and Management of Wilson’s Disease: A Practical Guide from the British Association for the Study of the Liver. Lancet Gastroenterol. Hepatol..

[107] Członkowska A., Litwin T., Dusek P., Ferenci P., Lutsenko S., Medici V., Rybakowski J.K., Weiss K.H., Schilsky M.L. (2018). Wilson Disease. Nat. Rev. Dis. Primers.

[108] Szeligowska J., Ilczuk T., Nehring P., Górnicka B., Litwin T., Członkowska A., Przybyłkowski A. (2022). Liver Injury in Wilson’s Disease: An Immunohistochemical Study. Adv. Med. Sci..

[109] Trache D., Hussin M.H., Hui Chuin C.T., Sabar S., Fazita M.R.N., Taiwo O.F.A., Hassan T.M., Haafiz M.K.M. (2016). Microcrystalline Cellulose: Isolation, Characterization and Bio-Composites Application—A Review. Int. J. Biol. Macromol..

[110] Nsor-Atindana J., Chen M., Goff H.D., Zhong F., Sharif H.R., Li Y. (2017). Functionality and Nutritional Aspects of Microcrystalline Cellulose in Food. Carbohydr. Polym..

[111] Ding D., Chen M., Li W., Luo Z., Xu Y., Zong W., Li W., Chen J. (2026). Development of a Gastrointestinal-Restricted Cellulose-Based Copper Sequestrant: Potential Application in Treating Wilson’s Disease. Int. J. Biol. Macromol..

[112] de Carvalho A.P.A., Értola R., Conte-Junior C.A. (2024). Nanocellulose-Based Platforms as a Multipurpose Carrier for Drug and Bioactive Compounds: From Active Packaging to Transdermal and Anticancer Applications. Int. J. Pharm..

[113] Dhiman S., Singh T.G., Rehni A.K. (2011). Transdermal Patches: A Recent Approch to New Drug Delivery System. Int. J. Pharmcy Pharm. Sci..

[114] Zhang L., Zhou X., Li X., Wang K., Zhou P., Ding W., Cui J., Qiao Y., Huang S., Luan C. (2025). Engineered Sulfonated Bacterial Cellulose Hydrogel with Dual Bioactive-Drug Delivery Functions for Precision Treatment of Psoriasis. Biomacromolecules.

[115] Gao X., Zhang H., Yan C., Wu J., Wang Y., Jiang M., Wang Y. (2025). Yunnan Baiyao-Enhanced Cellulose Nanofiber Composite Hydrogel Wearable Patch for Transdermal Drug Delivery and Anti-Freezing Applications. Int. J. Biol. Macromol..

[116] Pandya I., Joshi V., Pansuriya R., Raje N., Assiri M.A., Malek N. (2025). A Multifunctional IL@MOF Composite-Based Hydrogel for Enhanced Transdermal Drug Delivery of 5-Fluorouracil. J. Mater. Chem. B.

[117] Abraham A.M., Simon A., Anjani Q.K., Jiang Y., Adhami M., Dominguez-Robles J., Larraneta E., Donnelly R.F. (2025). Controlled Release of Amitriptyline via the Transdermal Route Using SmartReservoirs and Hydrogel-Forming Microneedles. Biomater. Adv..

[118] Tseng C.-S., Lu Z.-X., Lu W.T., Li Y.-C., Wu S.-Y. (2026). Cellulose-Based Aerogels for Microneedle Patch Applications. J. Taiwan. Inst. Chem. Eng..

[119] Zhang M., Wang Y. (2025). Citric Acid–Crosslinked Carboxymethyl Cellulose Hydrogel Microneedles Enable Gentle Loading and Rapid Transdermal Delivery of Insulin. Int. J. Pharm..

[120] Yousaf A., Ahmad Z., Mahmood A., Khan M.I., Akhtar M.F. (2025). Transdermal Co-Delivery of Sumatriptan Succinate and Naproxen Sodium via Dissolving Microneedle Patch. J. Pharm. Innov..

[121] Bahmani S., Khajavi R., Ehsani M., Rahimi M.K., Kalaee M.R. (2025). A Development of a Gelatin and Sodium Carboxymethyl Cellulose Hydrogel System for Dual-Release Transdermal Delivery of Lidocaine Hydrochloride. Int. J. Biol. Macromol..

[122] Li Y., Liu W., Wang Y., Lu S. (2025). Cellulose Based Nano-Scaffolds for Targeted Cancer Therapies: Current Status and Future Perspective. Int. J. Nanomed..

[123] Bao Z., Xue Y., Chen X., Wang Y., Wang J., Liu Y., Shao Z. (2025). Cellulose-Based Nanomaterials in Targeted Tumor Chemotherapy: A Comprehensive Review of Design, Delivery, and Clinical Potential. Ind. Crop. Prod..

[124] Ghosh S., Manna K., Chakraborty K., Dhara S., Pal S. (2025). Site-Specific Drug Delivery Using Injectable pH-Responsive Biopolymeric Hydrogel: Instant Crosslinking and Shear Thinning. ACS Appl. Polym. Mater..

[125] Han Z., Bao L., Yu Y., Zhao Y., Wang M., Sun Y., Fu M., Zhou Q., Liu W., Cui W. (2025). Injectable Short-Fiber Hydrogel with Fatigue Resistance and Antibacterial Properties for Synergistic Periodontitis Therapy. Chem. Eng. J..

[126] Kulkarni N., Rao P., Jadhav G.S., Kulkarni B., Kanakavalli N., Kirad S., Salunke S., Tanpure V., Sahu B. (2023). Emerging Role of Injectable Dipeptide Hydrogels in Biomedical Applications. ACS Omega.

[127] Zhou J., Zhang Z., Zhang Z., Wu T., Li H., Hao X., Liu X., Gong T., Liu D., Wei S. (2025). A Facile Dual-Drug Delivery System Using Cellulose-Based Microgel/Hydrogel for Enhanced Gastric Cancer Therapy. Colloids Surf. B Biointerfaces.

[128] Chatap V., Patel B., Patil T., Priyadarshi G., Shahid M., Syed R., Bagatharia S., Sahoo D.K., Patel A. (2025). Innovative Fabrication of Folic Acid-Conjugated Curcumin Cellulose Nanofibers for Targeted Lung Cancer Therapy. Cellulose.

